# Immune Checkpoint Inhibitors in Hepatocellular Carcinoma: Current Strategies and Biomarkers Predicting Response and/or Resistance

**DOI:** 10.3390/biomedicines11041020

**Published:** 2023-03-27

**Authors:** Filippo Pelizzaro, Fabio Farinati, Franco Trevisani

**Affiliations:** 1 Department of Surgery, Oncology and Gastroenterology, University of Padova, 35128 Padova, Italy; 2 Gastroenterology Unit, Azienda Ospedale-Università di Padova, 35128 Padova, Italy; 3 Department of Medical and Surgical Sciences, University of Bologna, 40126 Bologna, Italy; 4 Unit of Semeiotics, Liver and Alcohol-Related Diseases, IRCCS Azienda Ospedaliero-Universitaria di Bologna, 40138 Bologna, Italy

**Keywords:** liver cancer, immunotherapy, biomarkers, resistance, response, outcome

## Abstract

In recent years, immune checkpoint inhibitors (ICIs) have revolutionized the treatment of patients with hepatocellular carcinoma (HCC). Following the positive results of the IMbrave150 trial, the combination of atezolizumab (an anti-PD-L1 antibody) and bevacizumab (an anti-VEGF antibody) became the standard of care frontline treatment for patients with advanced stage HCC. Several other trials evaluated immunotherapy in HCC, demonstrating that ICIs-based regimens are currently the most effective treatment strategies and expanding the therapeutic possibilities. Despite the unprecedent rates of objective tumor response, not all patients benefit from treatment with ICIs. Therefore, in order to select the appropriate therapy as well as to correctly allocate medical resources and avoid unnecessary treatment-related toxicities, there is great interest in identifying the predictive biomarkers of response or resistance to immunotherapy-based regimens. Immune classes of HCC, genomic signatures, anti-drug antibodies, and patient-related factors (e.g., etiology of liver disease, gut microbiota diversity) have been associated to the response to ICIs, but none of the proposed biomarkers have been translated into clinical practice so far. Considering the crucial importance of this topic, in this review we aim to summarize the available data on tumor and clinical features associated with the response or resistance of HCC to immunotherapies.

## 1. Introduction

Primary liver cancer, which in 75–85% of cases is represented by hepatocellular carcinoma (HCC), is the sixth most commonly diagnosed cancer and the third leading cause of cancer-related death globally [1]. The magnitude of this relevant health problem is predicted to rise in the near future, with a 55% projected increase in new cases of liver cancer per year between 2020 to 2040 [2]. Despite the widespread use of surgical and locoregional therapies worldwide, it is estimated that 50–60% of all patients with HCC will be ultimately treated with systemic therapies [3]. For several decades, no systemic drugs were available given the lack of efficacy of the evaluated molecules. A milestone in the systemic treatment of patients with advanced HCC was the approval of sorafenib in 2008, following the demonstration of its efficacy in two randomized clinical trials (RCTs) [4,5]. With this achievement, the era of molecular-targeted therapies began. Tyrosine kinase inhibitors (TKIs) became the mainstay treatment of advanced HCC, holding the lead for more than a decade. These drugs as single agents demonstrated a clear survival benefit: a median overall survival (OS) of 11–14 months is achievable with first-line sorafenib and lenvatinib, while second-line regorafenib and cabozantinib grant an additional OS gain of 8–11 months [6]. Another breakthrough in the management of HCC was recently made, and currently we are witnessing the second revolution in the field of systemic therapies due to the demonstrated efficacy of immune-checkpoint inhibitors (ICIs). The positive results of the IMbrave150 trial, in which the combination of the anti-programmed death ligand 1 (PD-L1) atezolizumab and the anti-vascular endothelial growth factor A (VEGF-A) bevacizumab definitely demonstrated its superiority compared to sorafenib in patients with advanced stage HCC [7], are of the utmost importance. As a result of these findings, the more than 10-year TKIs-based treatment paradigm of advanced HCC has changed, and with this regimen a new benchmark for the median OS of approximately 19 months has been set [8]. The remarkable efficacy of immunotherapy in HCC was recently confirmed by the positive results of the HIMALAYA trial, in which the anti PD-L1 antibody durvalumab plus the anti-cytotoxic T lymphocyte antigen 4 (CTLA4) tremelimumab combination provided a statistically significant survival benefit compared to sorafenib in the first-line [9]. Moreover, this study also showed that durvalumab monotherapy is not inferior to sorafenib [9]. Considering the impressive survival benefit obtained with ICIs-based regimens, the treatment of advanced HCC has entered the era of immunotherapy. Therefore, as expected, research is currently dominated by clinical trials investigating ICIs combination therapies across all the stages of the disease, including adjuvant and neoadjuvant settings [3]. There is no doubt that ICIs have revolutionized the treatment of advanced stage HCC and that represents a very important step forward. However, there is still a relevant proportion of patients receiving ICIs who do not benefit from the treatment. Indeed, approximately 40% of HCC patients do not achieve disease control with ICIs due to primary resistance, and a similar proportion will experience disease progression after an initial response (secondary resistance) [10]. The identification and development of predictive biomarkers capable of accurately identifying patients who will benefit from ICIs (responders) is of the utmost importance to better understand and overcome mechanisms of resistance and, hence, to enable a precision medicine approach in HCC immunotherapy. This represents an unmet medical need and a research challenge, as no validated biomarkers capable of predicting the outcome of ICIs-treated patients are currently available in clinical practice. Nevertheless, some studies have tried to define potential biomarkers for immune-based treatment strategies in HCC [3]. This review aims to summarize the available evidence and recent advances on the identification of predictive biomarkers of response and resistance to immunotherapy in HCC patients.

## 2. Antitumor Immunity and Tumor Immune Escape

Our immune system can respond to infections and exogenous stimuli through a non-specific and quick response, the innate immunity, and a subsequently activated antigen-specific response that mediates the immune memory, called adaptative immunity [11].

**Figure 1:**
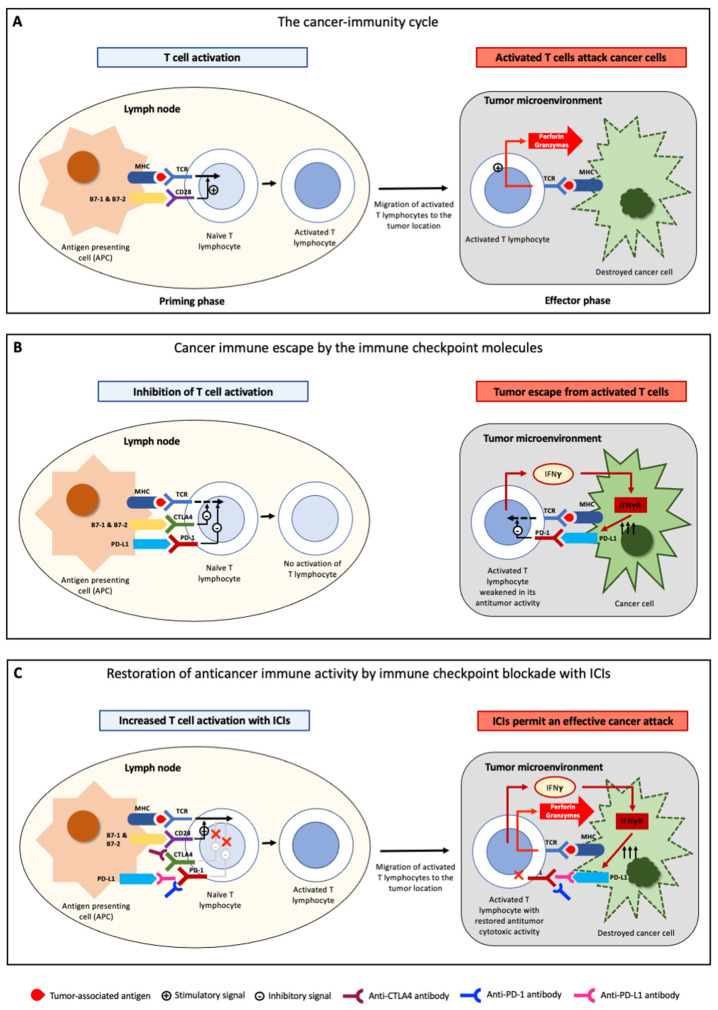
Mechanisms of anti-tumor immunity and cancer immune escape. (**A**) In lymph nodes, T lymphocytes are activated upon recognition of tumor-associated antigens presented by the major histocompatibility complex (MHC) expressed on antigen-presenting cells (APCs), in combination with a co-stimulatory signal through the B7-CD28 pathway. These activated T cells, in the tumor microenvironment, recognize tumor-associated antigens expressed on tumor cells and, in turn, attack malignant cells by releasing perforin and granzymes. (**B**) Immune checkpoint molecules (PD-1, PD-L1, CTLA4) induce cancer immune escape. T lymphocytes activation is suppressed by the B-7/CTLA4 pathway in lymph nodes, while the PD-1/PD-L1 pathway mediates immune escape in cancer cells. (**C**) Schematic mechanism of action of immune checkpoint inhibitors anti-PD-1, anti-PD-L1, and anti-CTLA4. Anti-CTLA4 restore T lymphocytes activation in lymph nodes and anti-PD-1/anti-PD-L1 antibodies allow an effective cancer attack. Adapted from Kudo, 2017 [26].

The activation of antitumor immunity is a complex process, in which several cell and lymphocyte populations are involved. The uncontrolled proliferation of cancer cells causes a high tumor mutational burden firstly leading to the activation of innate immunity. In particular, natural killer (NK) cells target cancer cells, causing their apoptosis and a release of tumor-associated antigens (TAAs), which are tumor proteins that the immune system recognizes as non-self. TAAs can be distinguished in non-mutated self-antigens and major histocompatibility complex (MHC)-I-restricted or MHC-II-restricted neoantigens, which arises as the result of non-synonymous somatic mutations [12,13,14,15,16]. Self-antigens are non-mutated proteins aberrantly expressed or overexpressed in tumor cells. Despite their contribution to tumor immunity, the primary targets of many antitumor immune responses are tumor-specific neoantigen peptides that are the result of somatic mutations in cancer genomes [13,14,17,18,19,20,21,22,23]. These neoantigens (mutated proteins), released by cancer cells in the tumor microenvironment (TME), are subsequently captured by antigen-presenting cells (APCs). The APCs then migrate to lymph nodes where they present neoantigens to T-cell receptors (TCR) on immature T lymphocytes through the MHC. However, antigen stimulation alone is not enough to activate naïve T lymphocytes, and an additional co-stimulatory signal is required. Indeed, T cells become fully activated after the binding between CD28 on T cells and CD80 or CD86 (named also B7-1 and B7-2, respectively) on APCs (priming phase) (Figure 1A). Upon returning from the lymph nodes to the tumor site, activated T cells recognize TAAs on the tumor cells and attack them via perforin and granzyme (effector phase) (Figure 1A) [11].

Although the immune system is able to recognize and destroy cancer cells, malignant cells can develop different mechanisms aimed at evading the host’s immune control, by reducing MHC complex expression on APCs or inhibiting the immune response (immune checkpoint molecules) [24,25]. Among these molecules, the most relevant are cytotoxic T-lymphocyte antigen 4 (CTLA4), programmed cell death protein 1 (PD-1) and its ligand (PD-L1), and lymphocyte-activation gene-3 (LAG-3). The fundamental role of these molecules is to counterbalance lymphocyte activation in physiological conditions, in order to avoid unnecessary tissue damage, chronic inflammation, and uncontrolled lymphocytic proliferation. In detail, CTLA4 exerts its activity solely within lymph nodes and is expressed constitutively on regulatory T cells (T_reg_) and transiently on several T cells in the early priming phase [26]. B7-1 and B7-2 (the molecules responsible for the co-stimulatory signal) also bind to CTLA4, with an affinity 10 times as strong as it is for CD28. As a consequence, the binding to CTLA4 is preferred over CD28, leading to the inhibition of T cells activation. CTLA4 acts as a brake on the activation and proliferation of T cells. This, in physiological conditions, prevents excessive immune responses by limiting unnecessary T cells activity, but it impairs anticancer immunity by hindering cancer antigens recognition (Figure 1B).

PD-1 is an immune co-inhibitory receptor expressed on T cells and on several other immune cells [26]. On T lymphocytes, PD-1 prevents antigen-specific activation through interactions with its ligands PD-L1 (expressed in dendritic cells and in cells from several other tissues) and PD-L2 (expressed only in dendritic cells). PD-1 is expressed selectively on T cells at the late activation stage, and its expression is particularly strong in effector T cells in peripheral tissues. In addition to a constitutive expression in normal peripheral tissues and on most immune cells during the initiation of immune response, PD-L1 is expressed also in the majority of cancer cells. By contrast, PD-L2 expression is selective and limited to APCs (and this explains why PD-L2 exerts a limited role in anticancer immunity). Upon recognition of TAAs presented by MHC on cancer cells, activated T lymphocytes release not only perforin and granzymes, but also interferon-γ (IFNγ) and other cytokines. As a consequence, cancer cells upregulate PD-L1, which through the binding to PD-1 on lymphocytes, leads to a weakening of the T cells’ response. The final result is an immune escape or immune tolerance (Figure 1B).

Tumor cells can take advantage of these mechanisms in order to avoid immune-mediated destruction, not only by expressing ligands activating the immune checkpoints (intrinsic resistance, elicited by tumor cells), but also by favoring the development of a tolerant TME through the recruitment of non-neoplastic cells expressing these ligands (extrinsic resistance) [27,28,29].

ICIs are monoclonal antibodies specifically designed to disrupt these ligand/receptor interactions, removing T cells inhibition and promoting their antitumoral cytotoxic activity (Figure 1C). The administration of anti-CTLA4 antibodies permits the interaction between CD28 and B7-1 (or B7-2) and allows the co-stimulatory signal, thus restoring an effective priming phase of T-lymphocytes. Similarly, antibodies against PD-1/PD-L1 release the brake mechanism provided by the interaction between PD-1/PD-L1 signaling, allowing T lymphocytes to remain active despite the inhibitory molecules expressed by the tumor.

## 3. Immune-Checkpoint Inhibitors—Mechanisms of Action

Immune-checkpoint molecules, key regulators of immune responses, are expressed not only by immune cells (T cells and APCs) but also in tumor cells. Physiologically, several molecules that act as inhibitory immune-checkpoint receptors (that naturally control and contain T cell activity) and co-stimulatory immune-checkpoint proteins (that enhance T cell expansion) are expressed on the cell surface [26]. Among these molecules, therapeutic targeting of PD-1/PD-L1 and CTLA4 constitutes the backbone of immunotherapy for solid cancers. Anti-PD-1 and anti-PD-L1 antibodies block PD-1/PD-L1 interaction, thus restoring the T lymphocyte effector function (Figure 1C) [30]. CTLA4 inhibition acts on the interaction between T cells and APCs during the priming phase, favoring the binding between B7-1/B7-2 co-stimulatory ligands and CD28 in lymphocytes, leading to activation of naïve CD4^+^ and CD8^+^ T cells (Figure 1C) [31]. Anti-CTLA4 therapy increases the number of active CD4^+^ and CD8^+^ T lymphocytes and decreased peripheral T cell clonality [32].

Even though molecular classification studies suggest that about 35% of HCCs have an immune-reactive TME [33,34] and PD-L1 expression is associated with a worst outcome [35], the activity of anti-PD-1/PD-L1 antibodies in HCC is modest compared with other malignancies. Indeed, objective radiologic responses are observed in only 15–20% of patients [36]. Although the causes of unresponsiveness to ICIs remain largely unclear, the existence of several molecules potentially involved in immune escape other than PD-1/PD-L1 and CTLA4 is a possible explanation. According to experimental and clinical data, anti-PD-1 therapy seems more active than anti-PD-L1. Recently, a network meta-analysis combining the data from 19 trials across multiple solid tumor types demonstrated higher efficacy of anti-PD-1 therapies [37]. The unfavorable pharmacokinetic properties of anti-PD-L1 antibodies [38] and the additional blockade of the PD-1/PD-L2 interaction by anti-PD-1 antibodies [39] could partly explain these findings.

## 4. Landscape of Immunotherapy for HCC

The results of the most important clinical trials exploring the results of immunotherapies in advanced-stage HCC are reported in Table 1. Several other trials investigating the role of ICIs as an adjuvant therapy after curative treatments, or as complementary therapy to intra-arterial therapies in locally advanced disease, are currently ongoing.

The era of immunotherapy for HCC patients started with studies evaluating the outcome of ICIs monotherapies. Based on the efficacy data of phase II trials, the FDA granted accelerated approval to the anti-PD-1 antibodies nivolumab and pembrolizumab in monotherapy for the second-line treatment of advanced stage HCC [40,41]. However, the confirmatory phase III studies of nivolumab vs. sorafenib in the frontline (CheckMate 459) [42] and pembrolizumab vs. placebo in the second-line (KEYNOTE-240) [43] failed to meet their primary OS endpoints (although the overall response rates and the safety profile was confirmed for both drugs). Despite being unsuccessful, phase III trials with ICIs monotherapies provided important information: (1) these drugs are safe and well-tolerated even in patients with HCC, most of whom have underlying liver cirrhosis; (2) the objective response rate is 15–18%, which is significantly higher than that achievable with TKIs [42,43]; (3) patients with an objective response have long survival [40]. In order to overcome the limitations of immunotherapy with a single agent in terms of response rate and patient survival, the combination of different drugs has been tested. In the following paragraphs, we briefly report established combination strategies (i.e., the combination of drugs tested in concluded phase III clinical trials).

### 4.1. Anti-PD1/PD-L1 in Association with Intravenous Anti-VEGF Agents

The IMbrave150 trial is a phase III clinical trial in which advanced HCC patients previously untreated were randomized to receive the combination of the anti-PD-L1 atezolizumab plus the anti-VEGF bevacizumab (atezo+beva) or sorafenib in a 2:1 ratio [7]. The combination of immunotherapy and antiangiogenic therapy is scientifically appealing considering that, through VEGF pathway inhibition, the immunosuppressive TME is converted in an immunostimulatory milieu consequently to the normalization of tumor vasculature and an immunomodulator effect [44,45]. The results of the IMbrave150 trial constitutes a milestone in HCC therapy, since atezo+beva was demonstrated to be significantly superior to sorafenib as a frontline systemic therapy, thus ending its long-lasting era [7]. Notably, the median OS in the atezo+beva group was 19.2 months (95% CI 17.0–23.7), the longest ever achieved with systemic therapy in HCC patients [8]. In addition, atezo+beva increased both progression-free survival (PFS) (6.9 months vs. 4.3 months, *p* < 0.0001) and objective response rate (ORR) (30% vs. 11%, *p* < 0.0001) compared to sorafenib [8]. Overall, the toxicity of the combination was manageable, with hypertension as the most commonly reported adverse event (AE) in 12% of cases [8]. Even though the proportion of both serious AEs (23% vs. 16%) and of patients that discontinued the treatment due to toxicity (22% vs. 12%) were slightly higher in the atezo+beva than in the sorafenib arm, the combination therapy was associated with a better quality of life [8].

The strategy of combining ICIs with anti-VEGF antibodies proved to be fruitful also in other studies, as the trial assessing the treatment based on anti-PD-1 antibody sintilimab combined with the anti-VEGF IBI305 (a bevacizumab biosimilar) demonstrated a statistically significant benefit compared to sorafenib in Chinese patients with HBV-related advanced HCC [46].

### 4.2. Combination of PD-1 and CTLA4 Inhibitors (Dual Checkpoint Blockade)

Since cancer cells can exploit different checkpoints to evade immune surveillance, the dual blockade of the CTLA4 and PD-1/PD-L1 pathway was tested in order to enhance the immune response against the tumor. This strategy has been adopted in the phase III HIMALAYA trial, that compared the association of the anti-PD-1 durvalumab plus the anti-CTLA4 tremelimumab vs. sorafenib in the first-line setting. The ICIs combination (STRIDE, single-tremelimumab regular-interval durvalumab) was administered as a single dose of tremelimumab plus durvalumab, followed by a scheduled infusion regimen of durvalumab. The primary outcome of this trial was the superiority in survival for STRIDE vs. sorafenib, while the secondary outcome was noninferiority of durvalumab alone vs. sorafenib [9]. The median OS was 16.4 months (95% CI, 14.2–19.6) with STRIDE, 16.6 months (95% CI, 14.1–19.1) with durvalumab, and 13.8 months (95% CI, 12.3–16.1) with sorafenib. The trial met both the primary and secondary endpoints, since the hazard ratio (HR) for STRIDE vs. sorafenib was 0.78 (96% CI, 0.65–0.93), and the durvalumab monotherapy was noninferior to sorafenib (HR 0.90; 96% CI, 0.75–1.03; noninferiority margin 1.08). Grade 3/4 treatment-emergent AEs occurred for 25.8% of patients with STRIDE, 12.9% with durvalumab, and 36.9% with sorafenib [9].

Another phase III trial comparing nivolumab (anti-PD-1) plus ipilimumab (anti-CTLA4) vs. sorafenib (Chackmate9DW) is currently ongoing. Nevertheless, the FDA already granted accelerated approval for this combination in second-line settings after the high overall response rate (31%) without significant safety issues demonstrated in the phase II study [47].

### 4.3. Anti-PD1/PD-L1 in Association with TKIs

Since ICIs and TKIs are both effective in treating HCC, it is conceivable that the combination of these drugs may be more effective than each monotherapy. Even though the simple summatory effect of two successful treatments cannot be excluded, a synergistic effect has been hypothesized considering that TKIs block the signaling from several growth factors and affect immune effectors [48]. Despite these premises, two phase III RCTs failed to demonstrate the superiority of the ICIs + TKIs combinations compared to the TKIs monotherapy [49,50]. In the COSMIC-312 trial, patients were randomized to receive sorafenib monotherapy, cabozantinib monotherapy, or the combination of cabozantinib plus atezolizumab (cabo+atezo) (1:1:2 ratio). While the median PFS was significantly superior in the combination arm compared to the sorafenib arm (6.8 vs. 4.2 months; HR 0.63, 99% CI, 0.44–0.91), the median OS was not statistically different between the two groups (15.4 vs. 15.5 months; HR 0.90, 96% CI, 0.69–1.18) [49]. Even though this trial was designed with OS and PFS as the dual primary endpoints, the lack of survival advantage in the same setting in which the combinations atezo+beva and durvalumab + tremelimumab have demonstrated a clear advantage over the same competitor (sorafenib) makes any further commercial progression of the combination cabo+atezo unlikely.

The LEAP-002 study is a phase III RTC that evaluated the efficacy of the combination lenvatinib plus pembrolizumab (lenva+pembro) compared to lenvatinib monotherapy. Lenva+pembro did not demonstrate to be significantly superior to lenvatinib monotherapy, since the primary endpoint (OS: HR 0.840, 95% CI, 0.708–0.997, *p* = 0.0227; PFS: HR 0.867, 95% CI, 0.734–1.024, *p* = 0.0466) did not meet pre-specified statistical significance [50]. In this study, the median OS in the lenvatinib monotherapy arm was the longest ever observed with this TKI in advanced HCC, and this may contribute to the failure of the combined therapy.

Conversely, the benefit of the combination of an anti-PD-1/anti-PD-L1 antibody with a TKI has been proved for the first time in a phase III trial enrolling mostly Asian patients with unresectable HCC. In this study, the combined treatment with camrelizumab (an anti-PD-1) plus rivoceranib (a TKI targeting VEGFR-2) demonstrated a statistically significant benefit compared to sorafenib in terms of both OS and PFS (HR 0.62, 95% CI, 0.49–0.80, and HR 0.52, 95% CI, 0.41–0.65, respectively) [51]. Moreover, ORR is significantly improved with the combination compared to sorafenib (25.4% vs. 5.9%; *p* < 0.0001). Despite the relatively high treatment-related adverse events rate observed with camrelizumab + rivoceranib (80.9% vs. 52.4% with sorafenib), this combination can represent a new first-line therapy option for patients with advanced HCC.

**Table 1:** Main results of clinical trials investigating immune checkpoint inhibitors for the treatment of advanced stage HCC patients.

Trial	Phase	Investigational Drug(s)	Comparator	Median OS (HR, 95% CI)	Median PFS (HR, 95% CI)	ORR	Treatment-Related Adverse Event Rates			
							Grade ≥ 3	Most Common (Grade 3–4)	Leading to Discontinuation	Leading to Death
*First-line setting*										
IMbrave150 [7,8]	III	Atezolizumab + Bevacizumab (*n* = 336)	Sorafenib (*n* = 165)	19.2 vs. 13.4 months (0.66, 0.52–0.85; *p* < 0.001)	6.9 vs. 4.3 months (0.65, 0.53–0.81; *p* < 0.001)	30% vs. 11% (*p* < 0.001)	43% vs. 46%	Hypertension (12%); AST increase (5%); proteinuria (4%)	22% vs. 12%	2% vs. <1%
ORIENT-32 [46]	III	Sintilimab + IBI305 (bevacizumab biosimilar) (*n* = 380)	Sorafenib (*n* = 191)	NE vs. 10.4 months (0.57, 0.43–0.75; *p* < 0.0001)	4.6 vs. 2.8 months (0.56, 0.46–0.70; *p* < 0.0001)	21% vs. 4% (*p* < 0.0001)	34% vs. 36%	Hypertension (14%); decreased platelet count (8%); proteinuria (5%)	14% vs. 6%	2% vs. 1%
CheckMate 459 [42]	III	Nivolumab (*n* = 371)	Sorafenib (*n* = 372)	16.4 vs. 14.7 months (0.85, 0.72–1.02; *p* = 0.075)	3.7 vs. 3.8 months (0.93, 0.79–1.10; *p* = ns)	15% vs. 7% (*p* = NR)	22% vs. 49%	AST increase (6%); fatigue (<1%); pruritus (<1%); diarrhea (<1%)	7% vs. 12%	1% vs. <1%
Cosmic 312 ^a^ [49]	III	Atezolizumab + Cabozantinib (*n* = 432)	Sorafenib (*n* = 217)	15.4 vs. 15.5 months (0.90, 0.69–1.18; *p* = 0.44)	6.8 vs. 4.2 months (0.63, 0.44–0.91; *p* = 0.0012)	11% vs. 4% (*p* = NR)	64% vs. 46%	Hypertension (9%); AST increase (9%); ALT increase (9%); PPES (8%)	14% vs. 8%	1% vs. <1%
HIMALAYA ^b^ [9]	III	Tremelimumab + Durvalumab (STRIDE) (*n* = 393)	Sorafenib (*n* = 389)	16.4 vs. 13.8 months (0.78, 0.65–0.93; *p* = 0.0035)	3.8 vs. 4.1 months (0.90, 0.75–1.05; *p* = NR)	20.1% vs. 5.1% (*p* = NR)	25.8% vs. 36.9%	Lipase increase (6.2%); AST increase (5.2%); diarrhea (4.4%); Hyponatremia (4.1%)	8.25 vs. 11.0%	2.3% vs. 0.8%
		Durvalumab (*n* = 389)	Sorafenib (*n* = 389)	16.4 vs. 16.6 months (0.86, 0.73–1.03; noninferiority margin 1.08)	3.7 vs. 4.1 months (1.02, 0.88–1.19; *p* = NR)	17.0% vs. 5.1% (*p* = NR)	12.9% vs. 36.9%	AST increase (6.7%); lipase increase (4.1%); ALT increase (3.1%)	4.1% vs. 11.0%	0% vs. 0.8%
LEAP-002 ^c^ [50]	III	Lenvatinib + Pembrolizumab (*n* = 395)	Lenvatinib (*n* = 399)	21.2 vs. 19.0 months (0.840, 0.708–0.997; *p* = 0.0227)	8.2 vs. 8.0 months (0.867, 0.734–1.024; *p* = 0.0466)	26.1% vs. 17.5% (*p* = NR)	62.5% vs. 57.5%	NR	NR	1.0% vs. 0.8%
RATIONALE-301 ^c^ [52]	III	Tislelizumab (*n* = 342)	Sorafenib (*n* = 332)	15.9 vs. 14.1 months (0.85, 0.71–1.02; *p* = NR) ^d^	2.2 vs. 3.5 months (1.10, 0.92–1.33; *p* = NR)	14.3% vs. 5.4% (*p* = NR)	48.2% vs. 65.4%	NR	10.9% vs. 18.5%	4.4% vs. 5.2%
NCT03764293 ^c^ [51]	III	Camrelizumab + Rivoceranib (Apatinib) (*n* = 272)	Sorafenib (*n* = 271)	22.1 vs. 15.2 months (0.62, 0.49–0.80; *p* < 0.0001)	5.6 vs. 3.7 months (0.52, 0.41–0.65; *p* < 0.0001)	25.4% vs. 5.9% (*p* < 0.0001)	80.9% vs. 52.4%	Hypertension (37.5%); Increased AST (16.5%); increased ALT (12.9%); PPES (12.1%)	24.3% (3.7% discontinued both drugs) vs. 4.5%	0.4% vs. 0.4%
*Second-line setting*										
KEYNOTE-224 [41]	II	Pembrolizumab (*n* = 104)	-	12.9 months	4.9 months	17%	26% (irAEs 4%)	Increased AST (7%); increased ALT (4%); fatigue (4%); hyperbilirubinemia (2%)	23%	1%
KEYNOTE-240 [43]	III	Pembrolizumab (*n* = 278)	Placebo (*n* = 135)	13.9 vs. 10.6 months (0.781, 0.611–0.998; *p* = 0.0238)	3.0 vs. 2.8 months (0.718, 0.570–0.904; *p* = 0.0022)	18.3% vs. 4.4% (*p* = 0.00007)	18.3% vs. 7.5% (irAEs 7.2%)	Increased AST (5.4%); increased ALT (3.6%)	17.2% vs. 9%	2.5% vs. 3.0%
KEYNOTE-394 ^c,e^ [53]	III	Pembrolizumab (*n* = 300)	Placebo (*n* = 153)	14.6 vs. 13.0 months (0.79, 0.63–0.99; *p* = 0.018)	2.6 vs. 2.3 months (0.74, 0.60–0.92; *p* = 0.0032)	12.7% vs. 1.3% (*p* = 0.00004)	14.4% vs. 5.9%	NR	NR	1% vs. 0%
CheckMate 040 [40,54]	I/II	Nivolumab (*n* = 214 ^f^)	- ^g^	15.6 months ^h^	4.0 months ^i^	14% ^g^	19% ^i^	Increased AST (4%); increased ALT (2%)	7% ^i^	0%
CheckMate 040 [47]	I/II	Nivolumab + ipilimumab (*n* = 50 in arm A ^j^)	- ^g^	22.8 months	NR	32%	53%	Increased AST (16%); increased lipase (12%); increased ALT (8%)	18%	2%
RESCUE [55]	II	Camrelizumab + Apatinib (*n* = 120)	- ^g^	NR	5.5 months	22.5%	76.7% (irAEs 7.4%)	Hypertension (34.2%); gGT increase (11.6%); neutropenia (11.1%); increased AST (10.5%); hyperbilirubinemia (10.5%)	9.2%	0.8%
NCT02519348 [56]	I/II	Durvalumab + Tremelimumab (*n* = 75 ^k^)	- ^g^	18.7 months	2.2 months	24%	37.8% (irAEs 12.2%)	Increased AST (12.2%); increased amylase (6.8%); increased lipase (6.8%)	10.8%	1.4%
NCT01008358 [57]	II	Tremelimumab (*n* = 21)	- ^g^	8.2 months	6.5 months	17.6%	NR	Increased AST (45%); hyponatremia (30%); increased ALT (25%); encephalopathy (15%)	NR	0%
NCT02989922 [58]	II	Camrelizumab (*n* = 217)	- ^g^	13.8 months	2.1 months	14.7%	22%	Increased AST (5%); hyperbilirubinemia (3%); neutropenia (3%)	4%	1%
RATIONALE-208 [59]	II	Tislelizumab (*n* = 249)	- ^g^	13.2 months	2.7 months	13%	15%	Increased AST (3%); increased ALT (1%); hyperbilirubinemia (1%); fatigue (1%)	5%	0%

Abbreviations: NE, not estimable; NR, not reported; ns, not statistically significant; PPES, palmar–plantar erythrodysesthesia; irAEs, immune-related adverse events; gGT, gamma glutamyl transferase; (^a^) The dual primary endpoints were progression-free survival in the first 372 patients randomly assigned to cabozantinib plus atezolizumab or sorafenib (the progression-free survival intention-to-treat [ITT] population), and overall survival in all patients randomly assigned to the combination treatment of cabozantinib plus atezolizumab or sorafenib. In this trial, *n* = 188 patients were assigned to single-agent cabozantinib treatment, and this treatment arm was compared to sorafenib. (^b^) In this trial, patients were randomized to receive: tremelimumab (one dose) plus durvalumab (STRIDE), durvalumab monotherapy, or sorafenib monotherapy. The primary endpoint was overall survival for STRIDE vs. sorafenib. Noninferiority for overall survival for durvalumab vs. sorafenib was a secondary endpoint. (^c^) Available only in form of abstract. (^d^) The trial met its primary endpoint of OS noninferiority between tislelizumab and sorafenib. (^e^) This trial, conducted in Asian patients previously treated in first-line, met its primary endpoint of OS superiority. (^f^) In the expansion phase of the trial, *n* = 214 patients were included. Among these patients, 145 (68%) previously received sorafenib. (^g^) No control arm (comparator) was present in this trial. (^h^) In sorafenib-experienced patients (*n* = 145). (^i^) These results refer to all the patients included in the dose expansion cohort of the study (*n* = 214) irrespective of prior treatment with sorafenib. (^j^) Nivolumab 1 mg/kg plus ipilimumab 3 mg/kg every 3 weeks (4 doses) followed by nivolumab 240 mg intravenously every 2 weeks. (^k^) Tremelimumab 300 mg plus durvalumab 1500 mg (one dose each during the first cycle) followed by durvalumab 1500 mg once every 4 weeks.

## 5. Biomarkers of Response and/or Resistance to Immune Checkpoint Inhibitors

Unfortunately, according to the RCTs data, only 10–30% of patients with advanced HCC demonstrate an objective response to ICIs (Table 1) and, when the disease control rate (including patients with stable disease) is considered, immunotherapy is effective in only 70% of patients. Therefore, 30–40% of patients do not benefit at all from ICIs, either due to primary (lack of initial response) or secondary resistance (development of resistance to treatment after initial response) [10]. The identification of biomarkers able to predict the response and resistance to ICIs is urgently needed in order to correctly allocate medical resources and avoid the exposition of non-responder patients to the treatment toxicity. Several studies have investigated both tumor tissue and blood-derived potential biomarkers of response and/or resistance to immunotherapy in advanced HCC (Table 2). The following paragraphs point out this topic.

### 5.1. Markers of Response and/or Resistance in Tumor Genome

#### 5.1.1. Tumor Mutational Burden

The immune response against tumor cells is mainly elicited by tumor-specific neoantigen peptides, produced as a result of somatic mutations in cancer genomes and recognized as non-self by the immune system. Mutations in driver genes or passenger mutations, as well as other genomic and post-transcriptomic alterations, can induce the formation of neoantigens [74,75]. The number of neoantigens has been positively correlated with tumor mutational burden (TMB) [76], and the overall mutational load is an objective but indirect measure of tumor immunogenicity, considered a predictor of tumor-specific T cell immunity [74]. Therefore, it is reasonable to hypothesize that TMB may affect the odds of generating immunogenic peptides and influence ICIs response [16]. Indeed, a high TMB has been associated with tumor immune infiltration, response to ICIs and survival in several solid tumor types in which TMBs of >10 mutations per megabase (mut/Mb) are common, such as melanoma and non-small-cell lung cancer (NSCLC) [77]. This association has been confirmed in several other tumor types [78], and a meta-analysis including the data of >1000 ICI-treated patients demonstrated that TMB was the strongest predictor of response to therapy [79]. Therefore, the association between mutational burden or neoantigen load and patient survival after immunotherapy is definitely established for a large share of cancer types [77]. By contrast, the utility of TMB as a genomic biomarker is limited in HCC [80,81], due to a low mutational burden (<3 mut/Mb) and a lack of association between high TMB and greater immune infiltration [33]. The presence of molecular alterations impairing the antigen presenting system such as loss of heterozygosity or hypermethylation of HLA, that hinder the recognition of cancer cells by the immune system, might explain these findings [62,82,83]. Indeed, in patients with HCC responding to ICIs, MHC II molecules are upregulated [84].

Microsatellite instability (MSI) is another condition associated with a high load of tumor-associated neoantigen peptides, which is in turn associated with high levels of intratumoral lymphocytes infiltration. For example, MSI-high (MSI-H) colon cancers, in which a high rate of non-synonymous single-nucleotide polymorphisms that lead to the expression of many neoantigens is present, show a higher intratumoral T cell content than MSI-low (MSI-L) colon cancers. The presence of infiltrating lymphocytes often predicts ICIs responsiveness because, although most of these cells exhibit markers of exhaustion, they can be reactivated by immune checkpoint antagonists [74]. In the HCC context, the utility of MSI evaluation as a genomic biomarker of ICIs response is limited by the low prevalence of MSI-H tumors (<1%) [85]. A recent study demonstrated that, among 755 patients with HCC undergoing ICIs, only 6 tumors (0.8%) were classifiable as TMB-high (TMB-H) and only one out of 542 tumors assessed for MSI (0.2%) was TMB-H and MSI-H [80]. In addition to the low prevalence of TMB-H and MSI-H HCCs, another limit in the applicability of these genomic biomarkers as reliable predictors of ICIs responsiveness is the lack of a standardized neoantigen prediction in human HCC [74].

Copy-number alterations (CNAs) influence tumor immune infiltration, and a high CNAs burden correlates with reduced immune cells infiltration, response to therapy, and survival in several types of cancers treated with ICIs [86]. In HCC, large-scale CNAs lead to the structural losses of genes involved in antigen presentation, suggesting that CNAs are partially responsible for immune desertification [34,62,63].

#### 5.1.2. Somatic Mutations

The somatic mutations of specific genes may confer to tumor cells the ability to evade immune surveillance or, conversely, make malignant cells more susceptible to immune system aggression [16]. In a mouse model of HCC, *CTNNB1* mutations and/or activation of the WNT—β-catenin signaling pathway interfere with dendritic cells (DCs) chemotaxis, promote immune escape, and favor resistance to ICIs through the downregulation of CCL5 expression [64]. Mutations causing *MYC* overexpression, present in approximately 50–70% of HCCs, result in PD-L1 translational upregulation [87]. *TP53* mutations, occurring in about 40% of HCCs, lead to p53 loss of function and promote the recruitment of immunosuppressive cells in the TME [34]. *ARID1A* mutations, a common driver event in HCC tumorigenesis, undermine mismatch repair thus leading to a higher TMB and an elevated number of tumor-infiltrating lymphocytes and PD-L1 expression [88]. Moreover, an *ARID1A*-deficient mice treatment with anti-PD-L1 antibodies reduced tumor burden and prolonged survival [88]. Nevertheless, *ARID1A* mutations are also able to downregulate IFNγ signaling by limiting chromatin accessibility [89]. The activation of CDK20 in HCCs promotes the recruitment of myeloid-derived suppressor cells (MDSCs) which potently inhibit autologous CD8^+^ T cells proliferation and activity [90]. Focal gains at chromosome 6p21, harboring the gene encoding for VEGF-A, result in an increased neoangiogenic activity and thereby facilitate an immunosuppressive TME [44,91].

The analysis of somatic mutations in cancer cells could be used as a marker of response/resistance to ICIs therapy. In an exploratory study including 31 HCC patients receiving ICIs, activating mutations affecting genes involved in the WNT-β-catenin signaling pathway were associated with lower disease control rates (0% vs. 53%), a shorter median PFS (2.0 vs. 7.4 months), and unfavorable survival outcomes (median OS 9.1 vs. 15.2 months) [65]. These results confirm the findings obtained in mice models, where β-catenin activation was associated with immune escape and resistance to anti-PD-1 therapy [64]. However, other studies have questioned the predictive potential of such alterations [84,92], making it necessary to await the results of future studies based on larger prospective cohorts.

#### 5.1.3. Gene Expression Profiling

Beyond mutational analysis, gene expression profiling is another potentially useful predictor of the response to ICIs. According to two retrospective studies, the activation of interferon signaling pathways and genes related to inflammation predict a favorable response to ICIs in patients with HCC [61,84]. The analysis of fresh and archival tumor samples from the Checkmate 040 trial allowed the identification of an inflammatory 4-genes signature able to predict an improved objective response rate (*p* = 0.05) and OS (*p* = 0.01) [61]. Very recently, a study evaluating the tumor samples from patients with advanced HCC showed that, among 28 patients treated with anti-PD-1 in the frontline, responders had upregulated IFNγ signaling and MHC II-related antigen presentation [84]. In addition, the authors demonstrated that a signature comprising 11 genes, including among others IFNγ signaling (STAT1, GBP1), antigen presentation (B2M, HLA-DRB5, HLA-DRA), and chemotaxis (CXCL9), was able to predict the prognosis. This signature was shown to predict response and survival also in a separate cohort of advanced HCC and in more than 240 patients with other solid cancers [84]. However, these very interesting findings require prospective confirmation in a larger cohort of patients with HCC.

### 5.2. Markers of Response and/or Resistance in Tumor Tissue

#### 5.2.1. Immunogenomic Classification in the Prediction of Response to ICIs

In recent years, some studies attempted to create a classification of HCC according to tumor immune features [3]. A study performed an immunogenomic analysis based on the clustering of immune-related gene-expression signatures in several cancers, identifying six immune subtypes. HCCs were predominantly classifiable in the “Inflammatory” and the “Lymphocyte depleted” subclasses [93]. The first study specifically investigating HCC samples was performed by Sia et al. in 2017 [33] and analyzed the gene expression pattern of inflammatory cells infiltrating TME. It identified an “Immune” class, accounting for about 25% of HCCs, characterized by markers of a significant inflammatory response; in particular, a high level of immune infiltration, high expression of immune checkpoints, and enrichment in the signatures involved in responsiveness to immunotherapy in other solid tumor types characterize this HCC group. In this “immune” class, two subtypes were identified according to distinct TME characteristics: (1) an “Immune Active” subclass, with high tumor infiltrating lymphocytes (TILs), increased CD8^+^ T cell and M1 macrophages infiltration, activated IFN signaling, and overexpression of adaptative immune response related genes; tumors pertaining to this subclass demonstrate favorable prognosis and better response to ICIs; and (2) an “Immune Exhausted” subclass, characterized by an activated stroma, increased infiltration of M2 macrophages and exhausted T cells, and high levels of TGFβ signaling [33].

Some refinements have been recently introduced in this classification after the identification of an “Inflamed” class of HCC [34]. This class, beyond including both the previously described “Immune” subclasses (“immune active” and “immune exhausted”), is expanded by the addition of the newly defined “Immune-like” subclass, which has features very similar to the “Immune active” subclass (high activation of interferon signaling, cytolytic activity, expression of immune-effector cytokines, and a more diverse T cell repertoire), but it is associated with the presence of *CTNNB1* mutations and increased WNT-β catenin signaling. Haber et al. [84] recently showed that HCCs belonging to the “Inflammatory class” are frequent among patients having clinical benefits from anti-PD-1/PD-L1 therapy. The proportion of inflamed HCCs is approximately 35%, a percentage higher than that reported in several malignancies (intrahepatic or extrahepatic cholangiocarcinoma, 11% [94,95]; small-cell lung cancer, 17% [96]; melanoma, 23% [97]), but lower than in others (squamous head and neck cancer, 40% [98]). The remaining 65% of “non-inflamed” HCCs are characterized by a low level of immune infiltrate, downregulation of immune checkpoint molecules, and low activation of interferon signaling. These tumors can be divided in two subclasses according to their immune escape mechanisms: (1) an “Intermediate” class, with a high level of chromosomal instability and, consequently, frequent deletions of immune-related genes (involved in IFN signaling or antigen presentation), and frequent *TP53* mutations; and (2) an “Excluded” class characterized by *CTNNB1* mutations, with increased WNT-β catenin signaling and *PTK2* overexpression due to gene amplification and promoter hypomethylation. The hallmark of the “Excluded” subclass is the presence of characteristics of immune desertification [34]. A schematic representation of HCC immune classes is shown in Table 3.

#### 5.2.2. PD-L1 Expression

In order to predict the response to anti-PD-1/anti-PD-L1 therapy, the evaluation of PD-L1 expression on tumor cells with immunohistochemistry (IHC) seems to be the ideal biomarker. Currently, PD-L1 IHC is used to decide whether to treat patients with NSCLC with anti-PD-1 therapy [99,100]. In particular, pembrolizumab is approved for NSCLC patients who are PD-L1^+^ (defined as PD-L1 on ≥50% of tumor cells in the first-line and ≥1% in the second-line). Nevertheless, despite multiple studies having demonstrated a positive correlation between PD-L1 expression and outcome in different tumor types [101,102,103,104], PD-L1 expression remains an imperfect predictor of ICIs response. In particular, evidence of a predictive role for PD-L1 immunostaining in HCC is elusive [3]. Calderaro et al. [35] in a series of 217 HCC patients showed that tumoral PD-L1 expression was significantly associated with markers of tumor aggressiveness (high serum alpha-fetoprotein [AFP], satellite nodules, macrovascular and microvascular invasion, and poor differentiation). Moreover, high PD-L1 expression on inflammatory cells of the tumor microenvironment correlated with poor prognostic features, such as high AFP levels, macrovascular invasion, poor differentiation, and high PD-1 expression [35]. In 174 patients included in the CheckMate 040 dose-expansion cohort, PD-L1 expression was associated with a better response to the study drug. In particular, 9/34 patients (26%) with PD-L1 expression on ≥1% of tumor cells had an objective response to nivolumab compared to 26/140 patients (19%) with PD-L1 expression <1% [40]. In addition, using fresh and archival tumor samples from dose-escalation and dose-expansion phases of the same study, the median OS was significantly longer in patients with tumor PD-L1 ≥1% than in those with PD-L1 <1% (28.1 vs. 16.6 months, *p* = 0.03) [61]. However, conflicting evidence on the predictive role of PD-L1 expression comes from the phase II KEYNOTE-224 trial, in which pembrolizumab was evaluated in the second-line [41]. In this study the association between the clinical efficacy of treatment and PD-L1 expression, defined as a combined positive score (number of PD-L1 positive tumor or immune cells divided by the total number of viable tumor cells) and as a tumor proportion score (percentage of viable tumor cells with partial or complete staining for PD-L1 relative to all viable cells), was assessed in 52/104 patients. A combined positive score >1% was associated with higher ORR (*p* = 0.021) and favorable PFS (*p* = 0.026), while a tumor proportion score >1% was not predictive of clinical benefits (*p* = 0.088 and *p* = 0.096, respectively). Moreover, even patients with low or undetectable PD-L1 tumor expression may experience durable clinical benefits when treated with ICIs [105]. Among the reasons potentially explaining these contradictory results on PD-L1 predictivity, the use of different detection assays, inter-assay and/or inter-observer heterogeneity, as well as non-standardized criteria and cut-offs defining positivity, may have played a role [16,106]. Therefore, the evaluation of PD-L1 expression is not a sufficient standalone biomarker for therapeutic decisions in clinic practice.

In addition to their expression levels, different isoforms of immune checkpoint inhibitor molecules can also be associated with resistance to immunotherapy. Δ42PD-1 is an alternatively spliced isoform of PD-1 lacking a 42-nucleotide region (equivalent to 14 amino acids) in exon 2 [107]. Unlike PD-1, this isoform does not bind to PD-L1 or PD-L2, and is not recognized by PD-1-specific monoclonal antibodies [107]. Δ42PD-1 mRNA was found differentially expressed in various immune-related cells (higher expression in monocytes, macrophages and NK cells; lower expression on B cells, CD4^+^ or CD8^+^ T cells, and dendritic cells) and it could induce the production of several proinflammatory cytokines (TNFα, IL-6, and Il-1β) from human peripheral blood mononuclear cells and murine dendritic cells [107]. A recent study evaluated the role of Δ42PD-1 in HCC progression and resistance to nivolumab and pembrolizumab [68]. The authors demonstrated that, compared to PD-1^+^ T cells, tumor-infiltrating Δ42PD-1^+^ T cells exhibit transcriptomic features of immune exhaustion and correlate positively with HCC severity. In addition, patients treated with anti-PD-1 antibodies (n = 28, nivolumab; n = 5, pembrolizumab) showed effective PD-1 blockade while non-responders displayed increased frequencies of Δ42PD-1^+^ T cells over time. The Δ42PD-1 isoform was not only associated with ICIs resistance, but it could also represent a therapeutic target. Indeed, in three murine models of HCC, anti-Δ42PD-1 antibodies inhibited tumor growth [68].

#### 5.2.3. Tumor-Infiltrating Lymphocytes and Other Immune Cells

It is commonly believed that ICIs, in particular anti-PD1 and anti-PD-L1, act by reinvigorating the pre-existing tumor immune response [108,109,110]. Therefore, the density of TILs within a tumor may be another potential predictor of the clinical effectiveness of this treatment. Such an assumption is further supported considering that, in some tumors, TILs density is a strong positive prognostic indicator regardless of ICIs therapy [111]. Moreover, not only the simple evaluation of TILs density, but also TILs phenotype and lymphocytes localization within a tumor, may provide prognostic information. Indeed, in patients treated with anti-PD-1, TILs density measured by IHC at the invasive margin of the tumor, and not central infiltration, was strongly associated with the response [109]. Even in HCC patients, tumor T cell infiltration within the tumor is predictive of response [34,66].

Beyond lymphocytes, many other immune cells infiltrating the tumor may affect the efficacy of ICIs. As already mentioned, HCCs with increased T-cell infiltration and higher expression of cytotoxic markers, immune-cell homing genes (CXCL9 and CXCL10), and IFNγ signaling (immune-active tumors) show a better response to anti-PD-1 monotherapy [34,61]. By contrast, some immune traits, such as the infiltration of CD4^+^ regulatory T cells or immunosuppressive macrophages (M2 vs. M1 polarization) and increased stromal activation with increased cancer-associated fibroblasts [34] are associated with resistance to immunotherapy. A recent study evaluated the ability of baseline serum concentrations of 59 cytokines and pretreatment tissue 11 immune cell markers to predict the clinical benefit in 33 patients treated with sintilimab + bevacizumab [66]. Patients with a high density of proinflammatory (M1) macrophages in the tissue and high serum concentration of CD137 at the baseline obtained a clinical benefit from the combination, defined as disease control at 12 weeks. In particular, a high density of M1 macrophages in the baseline tumor microenvironment was associated with better OS and PFS [66]. Despite being interesting, these results should be interpreted with caution, since the definition of clinical benefit used in this study is not considered a strong endpoint, and the proposed markers require further validation in independent and larger patient cohorts.

### 5.3. Markers of Response and/or Resistance Associated to the Host

#### 5.3.1. Role of Etiology in the Prediction of Response to Immune Checkpoint Inhibitors

Specific clinical features can influence the efficacy of immunotherapies in patients with HCC, and one of them is the etiology of the underlying liver disease. HCC etiologies (viral as compared to non-viral HCC) contribute differently in shaping the immune TME and, consequently, modulate the response to ICIs [3]. NASH-HCCs might be less responsive to immunotherapy, probably owing to NASH-related aberrant T cell activation causing tissue damage and impaired immune surveillance. Indeed, an increased intrahepatic infiltration of exhausted and unconventionally activated CD8^+^PD1^+^ T cells has been demonstrated in both mouse and human with NASH [67,112]. These CD8^+^PD1^+^ T cells exert cytotoxic activity against hepatocytes, resulting in necroinflammation. Moreover, the auto-aggressive behavior of intratumoral CD8^+^PD1^+^ T cells results in a loss of tumor surveillance function and in the development of a pro-tumorigenic microenvironment [112]. This auto-aggressive behavior of non-viral HCCs, and of particularly NASH-related HCCs, makes tumors less responsive to ICIs than virus-related ones [67]. In addition, immunosuppressive CXCR2^+^ neutrophils have been recently associated with resistance to anti-PD-1 therapy in NASH-HCC patients, while CXCR2 antagonization resulted in restoration of the response to ICIs [113].

A recent systematic review summarized the results of 49 phase III trials across all stages of HCC and investigated the relationship between etiology and outcome with various systemic therapies [60]. A meta-analysis of three trials assessing immunotherapies (CheckMate-459, IMbrave150, and KEYNOTE-240 for a total of 1656 patients) demonstrated that patients with a virus-related HCC treated with ICIs had a significantly greater OS benefit compared to the control group (HR = 0.64, 95% CI, 0.50–0.83). Instead, in non-viral patients, the survival of ICIs-treated patients was not significantly superior to that of the control group (HR = 0.92, 95% CI, 0.77-1.11) [60]. Remarkably, the clinical benefit provided by ICIs was similar in HBV- and HCV-related HCC. Moreover, a separate metanalysis of five trials (REACH, REACH-2, METIV-HCC, CELESTIAL, and JET-HCC for a total of 2083 patients) did not show an impact of etiology on outcome of TKIs/anti-VEGF, as no differences in efficacy were found between the viral and non-viral HCCs (*p* = 0.88) [60]. Results similar to those of meta-analyses have been found in a large retrospective study including almost 1000 patients with non-viral HCC in whom, after propensity score matching, lenvatinib provided a greater benefit in OS and PFS than atezo+beva [72].

However, differential clinical outcomes according to etiology have not been consistently reported across all immunotherapy RCTs [7,9,47], suggesting that the modulation of ICIs response by etiology is only partially unraveled. Moreover, although intriguing, data on this topic come from post-hoc analyses of phase III trials or retrospective studies and, as such, can be considered as “generating hypothesis” indications, providing a rationale to stratify patients by etiology in future trials but, for now, not supporting the choice of therapies other than ICIs for patients with non-viral HCCs in clinical practice.

#### 5.3.2. The Modulatory Role of Microbiota

Several studies demonstrated the existence of dysbiosis (an altered composition of gut microbiota) in different stages of chronic liver disease and in HCC patients [114,115]. Moreover, strong pre-clinical evidence demonstrate that HCC development in the setting of chronic liver diseases is promoted by the gut microbiota, probably through microbic metabolites and pathogens-associated molecular patterns (PAMPs) [116].

The gut microbiota has a role in the conversion of primary to secondary luminal bile acids, and alterations in the composition of normal commensal gut bacteria lead to the accumulation of primary bile acids and, in turn, an immunosurveillance suppression. Indeed, high levels of primary bile acids causes the secretion of CXCL16 from liver sinusoidal endothelial cells and the consequent recruitment of antitumor CXCR6+ NKT cells [117]. In addition, secondary bile acids (in particular deoxycholic acid) have been demonstrated to induce senescence of hepatic stellate cell, resulting in the release of cytokines that promote HCC development [118]. The role of gut microbiota in modulating systemic immunity, as well as influencing immunotherapy response and immune effects of chemotherapy, is now broadly accepted. In particular, microbial diversity is a host trait that can modulate the clinical response to immunotherapy-treated HCC patients [71]. Moreover, the dynamic variation of the gut microbiome characteristics may provide an early prediction of the outcomes of immunotherapy. These insights into the role of microbiota in modulating the response to ICIs, together with the evidence coming from pre-clinical studies on HCC prevention through the modulation of gut microbiota with antibiotic treatment [116], provide the rationale for evaluating the combination of immunotherapies and antibiotics or probiotics in clinical trials. Only preliminary experiences with this combination in the treatment of solid cancers are currently available. In a phase I trial, 30 patients with metastatic renal cell carcinoma were randomize to receive nivolumab + ipilimumab with or without the bifidogenic live bacterial product CBM588 [119]. PFS was longer (12.7 vs. 2.5 months; *p* = 0.001) and ORR was higher (58% vs. 20%; *p* = 0.06) in patients receiving nivolumab+ipilimumab with CMB588 compared to patients treated without the probiotic [119]. These preliminary data are extremely intriguing, but further studies better characterizing the effects of gut microbiome on hepatic immunity and specifically addressing the potential role of microbiota as biomarker of response in ICIs-treated patients are awaited [115].

### 5.4. Circulating Markers of Response and/or Resistance

In order to have a broad application in routine clinical practice, biomarkers should be assessed as less invasively as possible. Non-invasive biomarkers could be extremely useful for diagnostic or prognostic purposes. Therefore, the identification of whole blood-derived or serum-derived biomarkers through liquid biopsy is of great interest. In different tumor types, several blood-derived markers have been associated with the clinical outcomes of ICI treatment. In particular, total lymphocyte count, monocyte count, T cell clonality, neutrophil-to-lymphocyte ratio (NLR), relative eosinophil count, circulating T_reg_ cell levels, circulating monocytes or MDSCs, cytokine levels (IL-6, IL-8 and IL-10), and lactate dehydrogenase (LDH) activity have been evaluated as potential biomarkers [16,120,121,122]. Other potential predictive biomarkers associated with ICIs response may be identified through genomic analyses in blood-derived circulating-free DNA (cfDNA) [121]. In a study including 69 patients with 23 different cancer types (including HCC), the number of mutations detected in cfDNA was positively associated with ICIs’ response and survival [123]. However, limited data on cfDNA-derived biomarkers are currently available in patients with HCC receiving ICIs.

ICIs are monoclonal antibodies that can be recognized as non-self by the immune system, thus triggering the production of antidrug antibodies (ADAs). As for other malignancies, the ADAs relevance in HCC patients has aroused great interest. ADAs, reducing the drug bioavailability, decrease anti-tumor activity with detrimental effects on clinical outcomes. Therefore, the development of ADAs represents a potential mechanism for primary and acquired resistance. The probability of developing ADAs varies according the specific type of the drug administered, ranging from 54% with atezolizumab, to 10% with nivolumab, or 1.5% with pembrolizumab [3]. In the IMbrave150 trial, 28% of patients developed ADAs during treatment and less clinical benefit from the combination atezo+beva was demonstrated in ADAs positive compared to ADAs negative patients after 6 weeks of treatment (HR 0.93, 95% CI, 0.57–1.53, vs. HR 0.39, 95% CI, 0.26–0.60, respectively). This is probably due to a higher clearance of the drug in patients who developed ADAs [3]. In the HIMALAYA trial, among patients under immunotherapy, 1.7% developed neutralizing ADAs against durvalumab and 4.4% against tremelimumab [9]. However, the impact of ADAs development on outcomes was not reported. In a prospective cohort study enrolling 174 patients with HCC treated with atezo+beva (61 in the discovery cohort and 113 in the validation cohort), those with a robust ADA response (≥1000 ng/mL) after 3 weeks of therapy had a worse PFS and OS compared to the counterpart [70]. Moreover, compared with patients with low ADA levels, patients with high ADAs showed reduced serum atezolizumab concentration, impaired CD8^+^ T cell proliferation and had a decreased IFNγ and TNFα production from CD8^+^ T cell. All these data would confirm the potential role of ADAs as predictors of resistance to immunotherapy.

AFP is the most widely used circulating biomarker to define HCC aggressiveness. Its measurement has been studied as a tool for HCC early detection as well as to predict prognosis and response to locoregional and systemic therapies [124]. Baseline serum AFP may help in identifying those patients who will benefit most from molecular target therapies such as the VEGFR2 inhibitor ramucirumab, which demonstrated its effectiveness only in patients with AFP ≥ 400 ng/mL [125].

An AFP level drop during immunotherapy (the “AFP response”) has been investigated as a potential prognostic predictor. However, in different studies, the time point for AFP measurement during treatment (i.e., ranging from 1 week to 3 months) and the extent of AFP response to be considered relevant (i.e., a reduction of 20–50% compared with the baseline value) were chosen arbitrarily [126]. Recently, the utility of AFP decrease during atezo+beva treatment in predicting the response to treatment was evaluated using data from a phase Ib trial including 58 patients [69]. An AFP decrease ≥75% at week 6 was proposed in order to identify responders, with a sensitivity of 0.71 and a specificity of 0.91. The AFP increase of ≤10% was able to distinguish patients with disease control from primary progressors (sensitivity of 0.89 and specificity of 1). Even though both AFP cut-offs were associated with OS and PFS, such thresholds demonstrated a suboptimal accuracy (AUC 0.78 for response and AUC 0.66 for disease control) in a validation cohort of 150 patients treated with atezo+beva in the IMbrave150 trial [7]. The potential role of AFP changes as a useful indicator of response/resistance to ICI treatment requires further testing in properly powered cohorts with appropriate validation cohorts.

### 5.5. Clinical Markers of Immune Checkpoint Inhibitors Activity—Immune-Related Adverse Events

Patients receiving immunotherapy frequently experience adverse events (AEs), with severe (grade ≥ 3) treatment-related AEs occurring in 20–50% of those treated with anti-PD-1/anti-PD-L1 monotherapy [9,40,42,43,49]. Toxicity is even more frequent with ICIs combinations [9,49,50]. Typical toxicities of ICIs are immune-related AEs (irAEs), which are similar across different drugs of this class, and affect several organs, most frequently the skin, gastrointestinal tract, liver, lung, and endocrine glands [127]. Severe irAEs occur more frequently in patients treated with anti-CTLA4 monotherapy compared to those receiving anti-PD-1/anti-PD-L1 monotherapy (25% vs. 10–20%, respectively) [41,42,43,128]. When anti-PD-1/anti-PD-L1 and anti-CTLA4 antibodies are combined, immunotoxicity is cumulative, as demonstrated by the high percentage (around 50%) of patients requiring corticosteroids, an indicator of severe irAEs, in the phase II study evaluating nivolumab + ipilimumab [47]. Differently, when anti-PD-1/anti-PD-L1 antibodies are combined with anti-angiogenic agents, toxicities are addictive and mostly non-overlapping [7,50].

Interestingly, as found for TKI treatments [129], the development of irAEs heralds greater clinical benefit [73]. In particular, the development of treatment-related AEs graded ≥ 2 was independently associated with longer OS and PFS in a derivation cohort of HCC patients treated with ICIs therapy as a part of clinical trials submitted to FDA [73]. In the same study, these results were confirmed in an independent validation multicenter cohort. Therefore, the development of irAEs probably reflects a higher drug-induced immune activation directed against the tumor. However, when also considering the conflicting results observed in other tumor types [127], the association between irAEs emergence and higher ICIs efficacy remains to be firmly established in HCC patients.

## 6. Conclusions

ICIs have opened a new era in the therapeutic management of advanced stage HCC [3]. In comparison to TKIs, that dominated in the systemic treatment of liver cancer for more than a decade, ICIs are better tolerated and are able to provide higher rates of durable radiological objective response. The IMbrave150 trial [7,8], which lead to the approval of the use of atezo+beva combination in clinical practice, is currently the benchmark in the treatment of advanced stage HCC. Several other trials have investigated various combination of immunotherapy with very promising results (Table 1) and several more are yet to come in the near future, making it complex to navigate between the growing different therapeutic possibilities. Therefore, in order to guide the selection of appropriate therapy, to correctly allocate medical resources, and to avoid toxicities from unnecessary treatment, the availability of reliable biomarkers of response and/or resistance to ICIs is crucial. Several biomarkers have been recently investigated, but none of them have been translated into clinical practice so far. Moreover, to add complexity to this difficult issue, it is unlikely that a single biomarker will be sufficient in predicting the clinical benefit provided by ICIs and it probably will not have the same prognostic value for all ICI treatments and combinations. Indeed, patients who benefit from atezo+beva treatment are probably not the same patients who are likely to benefit from an ICI-TKI combination or an ICI-ICI combination. Each ICI (or combination) has a peculiar immunomodulatory effect and, probably, a target patient population who most likely benefit, as well as the most appropriate biomarker of response/resistance [3]. Moreover, in the prediction of ICIs clinical benefit, not only tumor-related but also patient-related factors (e.g., etiology of liver disease and gut microbiota diversity) should be considered [60,67,71]. Therefore, a single marker cannot take into account all these variables. Considering the available evidence, we are not yet close to having a suitable predictive model for ICIs efficacy. The complexity of the immune system and of tumor-host interaction does not make this task easy, although it is of crucial importance. Further translational research efforts are definitely needed to develop the biomarkers and predictive models that can identify responders to immunotherapy regimens.

## Figures and Tables

**Table 2:** Biomarkers of response to immune checkpoint inhibitors in advanced HCC.

Response	Refs	Resistance	Ref
*Biomarkers of response/resistance in tumor genome*			
Activation of IFN signaling pathways 4-genes signature 11-genes signature	[60,61]	Copy number alterations (CNA)—loss of heterozygosity of HLA alleles CTNNB1 mutations Activation of WNT/β-catenin pathway	[34,62,63] [64] [64,65]
*Biomarkers of response/resistance in tumor tissue*			
↑ T cell infiltration M1 macrophage polarization ↑ cytotoxicity *INFγ*, *PRF1*, *GZMB*, *TBX21*, *KLRK1* ↑ Immune cell homing *CXCL9*, *CXCL10* Antigen presentation—dendritic cells PD-L1 expression in cancer cells and/or immune cells	[34,66] [8,33,34] [34] [34,61] [34] [35,40,41,61]	↑ cancer-associated fibroblast ↑ stromal activation M2 macrophage polarization ↑ CD8+ PD1+ T cells *CCL5*, *CCL3*, *GZMK*, *GZMA*, *CXCR6* ↑ CD4 T_reg_ cells ↑ Δ42PD-1^+^ tumor-infiltrating T cells	[34] [33,34] [33,34] [67] [34] [68]
*Biomarkers of response/resistance in blood*			
↑ Baseline CD137 ↓ AFP during treatment	[66] [69]	Antidrug antibodies (ADAs)	[3,70]
*Biomarkers of response/resistance associated to the host*			
Gut microbiota diversity	[71]	Etiology—*NASH*	[60,67,72]
*Clinical markers of immunotherapy activity*			
Development of immune-related adverse events	[73]		

↑ = increase; ↓ = decrease.

**Table 3:** Schematic representation of histopathological and molecular features associated with immune classes in HCC (Montironi C. et al., 2023 [34]).

	Inflamed Class (~35%)			Non-Inflamed Class (~65%)		
	Active	Exhausted	Immune-Like	Intermediate	Excluded	
Tumor immune microenvironment	Immune infiltrate					
	+ + + +	+ + + −	+ + + −	+ − − −	− − − −	
	↑ TIL/TLS abundance			↓ TIL/TLS abundance		
	↑ CD8^+^ T cells and M1 macrophages	↑ M2 macrophages and exhausted T cells	↑ CD8^+^ T cells and M1 macrophages	↑ M2 macrophages and T_reg_ cells		
	↑ T cells/nucleated cells fraction, polyclonal T cell expansion and diverse TCR repertoire		Oligoclonal expansion	↓ T cells/nucleated cells fraction and diverse TCR repertoire		
	↑ immune-checkpoint molecules/CD8			↓ immune-checkpoint molecules/CD8		
	↑ PD-L1	↑ PD-L1	↑ LAG3 ↑ CTLA4	↓ LAG3 ↓ CTLA4	↓ CD8 ↓ PD-L1	
	↑ IFNγ, GZMB, PRF1, PD-1 signaling			↓ IFNγ, GZMB, PRF1, PD-1 signaling		
	Liquid Biopsy-based cytokine prediction of the inflamed class			↓ CXCL9, CXCL10, CXCL11, CCL5		
Molecular characterization		↑ TGFβ	↑ Wnt-β catenin		↑ Wnt-β catenin	↑ PTK2
	↑ Hoshida S1		↑ Hoshida S2	↑ Hoshida S2/3		
	↑ Chiang IFN		↑ Chiang CTNNB1	↑ Chiang Poly7	↑ Chiang CTNNB1	
Genomic characterization	Chromosomal aberrations					
	− − − −	+ − − −	+ + − −	+ + + −	+ + + +	
				↑ deletions at 16p13.13, 4q21.1, 4q35.1	Amplification 8q24.3	
			↑ CTNNB1 mutation	↑ TP53 mutation	↑ CTNNB1 mutation	
Epigenetic features	192 immune-related genes differentially methylated		Hypomethylation of HLA-I related genes		Hypomethylation of PTK2	

Abbreviations: TIL; tumor-infiltrating lymphocytes; TLS, tertiary lymphoid structures; TCR, T cell receptor; PD-L1, programmed death-ligand 1; LAG3, lymphocyte-activation gene 3; CTLA4, cytotoxic T lymphocyte antigen 4; INFγ, interferon γ; GZMB, Granzyme B; PRF1, perforin 1; PD-1, programmed death protein 1; CXCL9, chemokine ligand 9; CXCL10, chemokine ligand 10; CXCL11, chemokine ligand 11; CCL5, chemokine ligand 5; TGFβ, transforming growth factor β; CTNNB1, catenin beta 1; T_reg_, T regulatory cells. ↑ = increase; ↓ = decrease.

## Data Availability

Not applicable.

## References

[1] Sung H., Ferlay J., Siegel R.L., Laversanne M., Soerjomataram I., Jemal A., Bray F. (2021). Global Cancer Statistics 2020: GLOBOCAN Estimates of Incidence and Mortality Worldwide for 36 Cancers in 185 Countries. CA Cancer J. Clin..

[2] Rumgay H., Arnold M., Ferlay J., Lesi O., Cabasag C.J., Vignat J., Laversanne M., McGlynn K.A., Soerjomataram I. (2022). Global burden of primary liver cancer in 2020 and predictions to 2040. J. Hepatol..

[3] Llovet J.M., Castet F., Heikenwalder M., Maini M.K., Mazzaferro V., Pinato D.J., Pikarsky E., Zhu A.X., Finn R.S. (2022). Immunotherapies for hepatocellular carcinoma. Nat. Rev. Clin. Oncol..

[4] Llovet J.M., Ricci S., Mazzaferro V., Hilgard P., Gane E., Blanc J.-F., de Oliveira A.C., Santoro A., Raoul J.-L., Forner A. (2008). Sorafenib in advanced hepatocellular carcinoma. N. Engl. J. Med..

[5] Cheng A.L., Kang Y.K., Chen Z., Tsao C.J., Qin S., Kim J.S., Luo R., Feng J., Ye S., Yang T.S. (2009). Efficacy and safety of sorafenib in patients in the Asia-Pacific region with advanced hepatocellular carcinoma: A phase III randomised, double-blind, placebo-controlled trial. Lancet Oncol..

[6] Llovet J.M., Montal R., Sia D., Finn R.S. (2018). Molecular therapies and precision medicine for hepatocellular carcinoma. Nat. Rev. Clin. Oncol..

[7] Finn R.S., Qin S., Ikeda M., Galle P.R., Ducreux M., Kim T.-Y., Kudo M., Breder V., Merle P., Kaseb A.O. (2020). Atezolizumab plus Bevacizumab in Unresectable Hepatocellular Carcinoma. N. Engl. J. Med..

[8] Cheng A.-L., Qin S., Ikeda M., Galle P.R., Ducreux M., Kim T.-Y., Lim H.Y., Kudo M., Breder V., Merle P. (2022). Updated efficacy and safety data from IMbrave150: Atezolizumab plus bevacizumab vs. sorafenib for unresectable hepatocellular carcinoma. J. Hepatol..

[9] Abou-Alfa G.K., Lau G., Kudo M., Chan S.L., Kelley R.K., Furuse J., Sukeepaisarnjaroen W., Kang Y.-K., Van Dao T., De Toni E.N. (2022). Tremelimumab plus Durvalumab in Unresectable Hepatocellular Carcinoma. NEJM Evid..

[10] De Lorenzo S., Tovoli F., Trevisani F. (2022). Mechanisms of Primary and Acquired Resistance to Immune Checkpoint Inhibitors in Patients with Hepatocellular Carcinoma. Cancers.

[11] Chen D.S., Mellman I. (2013). Oncology meets immunology: The cancer-immunity cycle. Immunity.

[12] Coulie P.G., Van den Eynde B.J., van der Bruggen P., Boon T. (2014). Tumour antigens recognized by T lymphocytes: At the core of cancer immunotherapy. Nat. Rev. Cancer.

[13] Tran E., Ahmadzadeh M., Lu Y.-C., Gros A., Turcotte S., Robbins P.F., Gartner J.J., Zheng Z., Li Y.F., Ray S. (2015). Immunogenicity of somatic mutations in human gastrointestinal cancers. Science.

[14] Tran E., Turcotte S., Gros A., Robbins P.F., Lu Y.-C., Dudley M.E., Wunderlich J.R., Somerville R.P., Hogan K., Hinrichs C.S. (2014). Cancer immunotherapy based on mutation-specific CD4+ T cells in a patient with epithelial cancer. Science.

[15] Sahin U., Derhovanessian E., Miller M., Kloke B.-P., Simon P., Löwer M., Bukur V., Tadmor A.D., Luxemburger U., Schrörs B. (2017). Personalized RNA mutanome vaccines mobilize poly-specific therapeutic immunity against cancer. Nature.

[16] Havel J.J., Chowell D., Chan T.A. (2019). The evolving landscape of biomarkers for checkpoint inhibitor immunotherapy. Nat. Rev. Cancer.

[17] Monach P.A., Meredith S.C., Siegel C.T., Schreiber H. (1995). A unique tumor antigen produced by a single amino acid substitution. Immunity.

[18] Robbins P.F., El-Gamil M., Li Y.F., Kawakami Y., Loftus D., Appella E., Rosenberg S.A. (1996). A mutated beta-catenin gene encodes a melanoma-specific antigen recognized by tumor infiltrating lymphocytes. J. Exp. Med..

[19] Dubey P., Hendrickson R.C., Meredith S.C., Siegel C.T., Shabanowitz J., Skipper J.C., Engelhard V.H., Hunt D.F., Schreiber H. (1997). The immunodominant antigen of an ultraviolet-induced regressor tumor is generated by a somatic point mutation in the DEAD box helicase p68. J. Exp. Med..

[20] Lennerz V., Fatho M., Gentilini C., Frye R.A., Lifke A., Ferel D., Wölfel C., Huber C., Wölfel T. (2005). The response of autologous T cells to a human melanoma is dominated by mutated neoantigens. Proc. Natl. Acad. Sci. USA.

[21] Gubin M.M., Zhang X., Schuster H., Caron E., Ward J.P., Noguchi T., Ivanova Y., Hundal J., Arthur C.D., Krebber W.-J. (2014). Checkpoint blockade cancer immunotherapy targets tumour-specific mutant antigens. Nature.

[22] Snyder A., Makarov V., Merghoub T., Yuan J., Zaretsky J.M., Desrichard A., Walsh L.A., Postow M.A., Wong P., Ho T.S. (2014). Genetic basis for clinical response to CTLA-4 blockade in melanoma. N. Engl. J. Med..

[23] Rizvi N.A., Hellmann M.D., Snyder A., Kvistborg P., Makarov V., Havel J.J., Lee W., Yuan J., Wong P., Ho T.S. (2015). Cancer immunology. Mutational landscape determines sensitivity to PD-1 blockade in non-small cell lung cancer. Science.

[24] Chambers C.A., Kuhns M.S., Egen J.G., Allison J.P. (2001). CTLA-4-mediated inhibition in regulation of T cell responses: Mechanisms and manipulation in tumor immunotherapy. Annu. Rev. Immunol..

[25] Liechtenstein T., Dufait I., Bricogne C., Lanna A., Pen J., Breckpot K., Escors D. (2012). PD-L1/PD-1 Co-Stimulation, a Brake for T cell Activation and a T cell Differentiation Signal. J. Clin. Cell. Immunol..

[26] Kudo M. (2017). Immune Checkpoint Inhibition in Hepatocellular Carcinoma: Basics and Ongoing Clinical Trials. Oncology.

[27] Benci J.L., Xu B., Qiu Y., Wu T.J., Dada H., Twyman-Saint Victor C., Cucolo L., Lee D.S.M., Pauken K.E., Huang A.C. (2016). Tumor Interferon Signaling Regulates a Multigenic Resistance Program to Immune Checkpoint Blockade. Cell.

[28] Koyama S., Akbay E.A., Li Y.Y., Herter-Sprie G.S., Buczkowski K.A., Richards W.G., Gandhi L., Redig A.J., Rodig S.J., Asahina H. (2016). Adaptive resistance to therapeutic PD-1 blockade is associated with upregulation of alternative immune checkpoints. Nat. Commun..

[29] Shin D.S., Zaretsky J.M., Escuin-Ordinas H., Garcia-Diaz A., Hu-Lieskovan S., Kalbasi A., Grasso C.S., Hugo W., Sandoval S., Torrejon D.Y. (2017). Primary Resistance to PD-1 Blockade Mediated by JAK1/2 Mutations. Cancer Discov..

[30] Ahn E., Araki K., Hashimoto M., Li W., Riley J.L., Cheung J., Sharpe A.H., Freeman G.J., Irving B.A., Ahmed R. (2018). Role of PD-1 during effector CD8 T cell differentiation. Proc. Natl. Acad. Sci. USA..

[31] Maker A.V., Attia P., Rosenberg S.A. (2005). Analysis of the cellular mechanism of antitumor responses and autoimmunity in patients treated with CTLA-4 blockade. J. Immunol..

[32] Agdashian D., ElGindi M., Xie C., Sandhu M., Pratt D., Kleiner D.E., Figg W.D., Rytlewski J.A., Sanders C., Yusko E.C. (2019). The effect of anti-CTLA4 treatment on peripheral and intra-tumoral T cells in patients with hepatocellular carcinoma. Cancer Immunol. Immunother..

[33] Sia D., Jiao Y., Martinez-Quetglas I., Kuchuk O., Villacorta-Martin C., Castro de Moura M., Putra J., Camprecios G., Bassaganyas L., Akers N. (2017). Identification of an Immune-specific Class of Hepatocellular Carcinoma, Based on Molecular Features. Gastroenterology.

[34] Montironi C., Castet F., Haber P.K., Pinyol R., Torres-Martin M., Torrens L., Mesropian A., Wang H., Puigvehi M., Maeda M. (2023). Inflamed and non-inflamed classes of HCC: A revised immunogenomic classification. Gut.

[35] Calderaro J., Rousseau B., Amaddeo G., Mercey M., Charpy C., Costentin C., Luciani A., Zafrani E.-S., Laurent A., Azoulay D. (2016). Programmed death ligand 1 expression in hepatocellular carcinoma: Relationship with clinical and pathological features. Hepatology.

[36] Ribas A., Wolchok J.D. (2018). Cancer immunotherapy using checkpoint blockade. Science.

[37] Duan J., Cui L., Zhao X., Bai H., Cai S., Wang G., Zhao Z., Zhao J., Chen S., Song J. (2020). Use of Immunotherapy with Programmed Cell Death 1 vs. Programmed Cell Death Ligand 1 Inhibitors in Patients with Cancer: A Systematic Review and Meta-analysis. JAMA Oncol..

[38] Kurino T., Matsuda R., Terui A., Suzuki H., Kokubo T., Uehara T., Arano Y., Hisaka A., Hatakeyama H. (2020). Poor outcome with anti-programmed death-ligand 1 (PD-L1) antibody due to poor pharmacokinetic properties in PD-1/PD-L1 blockade-sensitive mouse models. J. Immunother. Cancer.

[39] Yearley J.H., Gibson C., Yu N., Moon C., Murphy E., Juco J., Lunceford J., Cheng J., Chow L.Q.M., Seiwert T.Y. (2017). PD-L2 Expression in Human Tumors: Relevance to Anti-PD-1 Therapy in Cancer. Clin. Cancer Res..

[40] El-Khoueiry A.B., Sangro B., Yau T., Crocenzi T.S., Kudo M., Hsu C., Kim T.Y., Choo S.P., Trojan J., Welling T.H. (2017). Nivolumab in patients with advanced hepatocellular carcinoma (CheckMate 040): An open-label, non-comparative, phase 1/2 dose escalation and expansion trial. Lancet.

[41] Zhu A.X., Finn R.S., Edeline J., Cattan S., Ogasawara S., Palmer D., Verslype C., Zagonel V., Fartoux L., Vogel A. (2018). Pembrolizumab in patients with advanced hepatocellular carcinoma previously treated with sorafenib (KEYNOTE-224): A non-randomised, open-label phase 2 trial. Lancet Oncol..

[42] Yau T., Park J.-W., Finn R.S., Cheng A.-L., Mathurin P., Edeline J., Kudo M., Harding J.J., Merle P., Rosmorduc O. (2022). Nivolumab versus sorafenib in advanced hepatocellular carcinoma (CheckMate 459): A randomised, multicentre, open-label, phase 3 trial. Lancet Oncol..

[43] Finn R.S., Ryoo B.Y., Merle P., Kudo M., Bouattour M., Lim H.Y., Breder V., Edeline J., Chao Y., Ogasawara S. (2020). Pembrolizumab as Second-Line Therapy in Patients with Advanced Hepatocellular Carcinoma in KEYNOTE-240: A Randomized, Double-Blind, Phase III Trial. J. Clin. Oncol..

[44] Huinen Z.R., Huijbers E.J.M., van Beijnum J.R., Nowak-Sliwinska P., Griffioen A.W. (2021). Anti-angiogenic agents—Overcoming tumour endothelial cell anergy and improving immunotherapy outcomes. Nat. Rev. Clin. Oncol..

[45] Fukumura D., Kloepper J., Amoozgar Z., Duda D.G., Jain R.K. (2018). Enhancing cancer immunotherapy using antiangiogenics: Opportunities and challenges. Nat. Rev. Clin. Oncol..

[46] Ren Z., Xu J., Bai Y., Xu A., Cang S., Du C., Li Q., Lu Y., Chen Y., Guo Y. (2021). Sintilimab plus a bevacizumab biosimilar (IBI305) versus sorafenib in unresectable hepatocellular carcinoma (ORIENT-32): A randomised, open-label, phase 2–3 study. Lancet Oncol..

[47] Yau T., Kang Y.-K., Kim T.-Y., El-Khoueiry A.B., Santoro A., Sangro B., Melero I., Kudo M., Hou M.-M., Matilla A. (2020). Efficacy and Safety of Nivolumab Plus Ipilimumab in Patients with Advanced Hepatocellular Carcinoma Previously Treated with Sorafenib: The CheckMate 040 Randomized Clinical Trial. JAMA Oncol..

[48] Kudo M. (2018). Combination Cancer Immunotherapy in Hepatocellular Carcinoma. Liver Cancer.

[49] Kelley R.K., Rimassa L., Cheng A.-L., Kaseb A., Qin S., Zhu A.X., Chan S.L., Melkadze T., Sukeepaisarnjaroen W., Breder V. (2022). Cabozantinib plus atezolizumab versus sorafenib for advanced hepatocellular carcinoma (COSMIC-312): A multicentre, open-label, randomised, phase 3 trial. Lancet Oncol..

[50] Finn R.S., Kudo M., Merle P., Meyer T., Qin S., Ikeda M., Xu R., Edeline J., Ryoo B.-Y., Ren Z. (2022). LBA34 Primary results from the phase III LEAP-002 study: Lenvatinib plus pembrolizumab versus lenvatinib as first-line (1L) therapy for advanced hepatocellular carcinoma (aHCC). Ann. Oncol..

[51] Qin S., Chan L.S., Gu S., Bai Y., Ren Z., Lin X., Chen Z., Jia W., Jin Y., Guo Y. (2022). LBA35 Camrelizumab (C) plus rivoceranib (R) vs. sorafenib (S) as first-line therapy for unresectable hepatocellular carcinoma (uHCC): A randomized, phase III trial. Ann. Oncol..

[52] Qin S., Kudo M., Meyer T., Finn R.S., Vogel A., Bai Y., Guo Y., Meng Z., Zhang T., Satoh T. (2022). LBA36 Final analysis of RATIONALE-301: Randomized, phase III study of tislelizumab versus sorafenib as first-line treatment for unresectable hepatocellular carcinoma. Ann. Oncol..

[53] Qin S., Chen Z., Fang W., Ren Z., Xu R., Ryoo B.-Y., Meng Z., Bai Y., Chen X., Liu X. (2022). Pembrolizumab plus best supportive care versus placebo plus best supportive care as second-line therapy in patients in Asia with advanced hepatocellular carcinoma (HCC): Phase 3 KEYNOTE-394 study. J. Clin. Oncol..

[54] El-Khoueiry A.B., Melero I., Yau T.C., Crocenzi T.S., Kudo M., Hsu C., Choo S., Trojan J., Welling T., Meyer T. (2018). Impact of antitumor activity on survival outcomes, and nonconventional benefit, with nivolumab (NIVO) in patients with advanced hepatocellular carcinoma (aHCC): Subanalyses of CheckMate-040. J. Clin. Oncol..

[55] Xu J., Shen J., Gu S., Zhang Y., Wu L., Wu J., Shao G., Zhang Y., Xu L., Yin T. (2021). Camrelizumab in Combination with Apatinib in Patients with Advanced Hepatocellular Carcinoma (RESCUE): A Nonrandomized, Open-label, Phase II Trial. Clin. Cancer Res..

[56] Kelley R.K., Sangro B., Harris W., Ikeda M., Okusaka T., Kang Y.-K., Qin S., Tai D.W.-M., Lim H.Y., Yau T. (2021). Safety, Efficacy, and Pharmacodynamics of Tremelimumab Plus Durvalumab for Patients with Unresectable Hepatocellular Carcinoma: Randomized Expansion of a Phase I/II Study. J. Clin. Oncol..

[57] Sangro B., Gomez-Martin C., de la Mata M., Iñarrairaegui M., Garralda E., Barrera P., Riezu-Boj J.I., Larrea E., Alfaro C., Sarobe P. (2013). A clinical trial of CTLA-4 blockade with tremelimumab in patients with hepatocellular carcinoma and chronic hepatitis C. J. Hepatol..

[58] Qin S., Ren Z., Meng Z., Chen Z., Chai X., Xiong J., Bai Y., Yang L., Zhu H., Fang W. (2020). Camrelizumab in patients with previously treated advanced hepatocellular carcinoma: A multicentre, open-label, parallel-group, randomised, phase 2 trial. Lancet Oncol..

[59] Ren Z., Ducreux M., Abou-Alfa G.K., Merle P., Fang W., Edeline J., Li Z., Wu L., Assenat E., Hu S. (2022). Tislelizumab in Patients with Previously Treated Advanced Hepatocellular Carcinoma (RATIONALE-208): A Multicenter, Non-Randomized, Open-Label, Phase 2 Trial. Liver Cancer.

[60] Haber P.K., Puigvehí M., Castet F., Lourdusamy V., Montal R., Tabrizian P., Buckstein M., Kim E., Villanueva A., Schwartz M. (2021). Evidence-Based Management of Hepatocellular Carcinoma: Systematic Review and Meta-analysis of Randomized Controlled Trials (2002–2020). Gastroenterology.

[61] Sangro B., Melero I., Wadhawan S., Finn R.S., Abou-Alfa G.K., Cheng A.-L., Yau T., Furuse J., Park J.-W., Boyd Z. (2020). Association of inflammatory biomarkers with clinical outcomes in nivolumab-treated patients with advanced hepatocellular carcinoma. J. Hepatol..

[62] Dong L.-Q., Peng L.-H., Ma L.-J., Liu D.-B., Zhang S., Luo S.-Z., Rao J.-H., Zhu H.-W., Yang S.-X., Xi S.-J. (2020). Heterogeneous immunogenomic features and distinct escape mechanisms in multifocal hepatocellular carcinoma. J. Hepatol..

[63] Bassaganyas L., Pinyol R., Esteban-Fabró R., Torrens L., Torrecilla S., Willoughby C.E., Franch-Expósito S., Vila-Casadesús M., Salaverria I., Montal R. (2020). Copy-Number Alteration Burden Differentially Impacts Immune Profiles and Molecular Features of Hepatocellular Carcinoma. Clin. Cancer Res..

[64] Ruiz de Galarreta M., Bresnahan E., Molina-Sánchez P., Lindblad K.E., Maier B., Sia D., Puigvehi M., Miguela V., Casanova-Acebes M., Dhainaut M. (2019). β-Catenin Activation Promotes Immune Escape and Resistance to Anti-PD-1 Therapy in Hepatocellular Carcinoma. Cancer Discov..

[65] Harding J.J., Nandakumar S., Armenia J., Khalil D.N., Albano M., Ly M., Shia J., Hechtman J.F., Kundra R., El Dika I. (2019). Prospective Genotyping of Hepatocellular Carcinoma: Clinical Implications of Next-Generation Sequencing for Matching Patients to Targeted and Immune Therapies. Clin. Cancer Res..

[66] Zhang W., Gong C., Peng X., Bi X., Sun Y., Zhou J., Wu F., Zeng H., Wang Y., Zhou H. (2022). Serum Concentration of CD137 and Tumor Infiltration by M1 Macrophages Predict the Response to Sintilimab plus Bevacizumab Biosimilar in Advanced Hepatocellular Carcinoma Patients. Clin. Cancer Res..

[67] Pfister D., Núñez N.G., Pinyol R., Govaere O., Pinter M., Szydlowska M., Gupta R., Qiu M., Deczkowska A., Weiner A. (2021). NASH limits anti-tumour surveillance in immunotherapy-treated HCC. Nature.

[68] Tan Z., Chiu M.S., Yang X., Yue M., Cheung T.T., Zhou D., Wang Y., Chan A.W.-H., Yan C.W., Kwan K.Y. (2022). Isoformic PD-1-mediated immunosuppression underlies resistance to PD-1 blockade in hepatocellular carcinoma patients. Gut.

[69] Zhu A.X., Dayyani F., Yen C.-J., Ren Z., Bai Y., Meng Z., Pan H., Dillon P., Mhatre S.K., Gaillard V.E. (2022). Alpha-fetoprotein as a potential surrogate biomarker for atezolizumab + bevacizumab treatment of hepatocellular carcinoma. Clin. Cancer Res..

[70] Kim C., Yang H., Kim I., Kang B., Kim H., Kim H., Lee W.S., Jung S., Lim H.Y., Cheon J. (2022). Association of High Levels of Antidrug Antibodies against Atezolizumab with Clinical Outcomes and T-Cell Responses in Patients with Hepatocellular Carcinoma. JAMA Oncol..

[71] Zheng Y., Wang T., Tu X., Huang Y., Zhang H., Tan D., Jiang W., Cai S., Zhao P., Song R. (2019). Gut microbiome affects the response to anti-PD-1 immunotherapy in patients with hepatocellular carcinoma. J. Immunother. Cancer.

[72] Rimini M., Rimassa L., Ueshima K., Burgio V., Shigeo S., Tada T., Suda G., Yoo C., Cheon J., Pinato D.J. (2022). Atezolizumab plus bevacizumab versus lenvatinib or sorafenib in non-viral unresectable hepatocellular carcinoma: An international propensity score matching analysis. ESMO Open.

[73] Pinato D.J., Marron T.U., Mishra-Kalyani P.S., Gong Y., Wei G., Szafron D., Sharon E., Saeed A., Jun T., Dharmapuri S. (2021). Treatment-related toxicity and improved outcome from immunotherapy in hepatocellular cancer: Evidence from an FDA pooled analysis of landmark clinical trials with validation from routine practice. Eur. J. Cancer.

[74] Hou J., Zhang H., Sun B., Karin M. (2020). The immunobiology of hepatocellular carcinoma in humans and mice: Basic concepts and therapeutic implications. J. Hepatol..

[75] Michler T., Kosinska A.D., Festag J., Bunse T., Su J., Ringelhan M., Imhof H., Grimm D., Steiger K., Mogler C. (2020). Knockdown of Virus Antigen Expression Increases Therapeutic Vaccine Efficacy in High-Titer Hepatitis B Virus Carrier Mice. Gastroenterology.

[76] Liu D., Schilling B., Liu D., Sucker A., Livingstone E., Jerby-Arnon L., Zimmer L., Gutzmer R., Satzger I., Loquai C. (2019). Integrative molecular and clinical modeling of clinical outcomes to PD1 blockade in patients with metastatic melanoma. Nat. Med..

[77] Samstein R.M., Lee C.-H., Shoushtari A.N., Hellmann M.D., Shen R., Janjigian Y.Y., Barron D.A., Zehir A., Jordan E.J., Omuro A. (2019). Tumor mutational load predicts survival after immunotherapy across multiple cancer types. Nat. Genet..

[78] Yarchoan M., Hopkins A., Jaffee E.M. (2017). Tumor Mutational Burden and Response Rate to PD-1 Inhibition. N. Engl. J. Med..

[79] Litchfield K., Reading J.L., Puttick C., Thakkar K., Abbosh C., Bentham R., Watkins T.B.K., Rosenthal R., Biswas D., Rowan A. (2021). Meta-analysis of tumor- and T cell-intrinsic mechanisms of sensitization to checkpoint inhibition. Cell.

[80] Ang C., Klempner S.J., Ali S.M., Madison R., Ross J.S., Severson E.A., Fabrizio D., Goodman A., Kurzrock R., Suh J. (2019). Prevalence of established and emerging biomarkers of immune checkpoint inhibitor response in advanced hepatocellular carcinoma. Oncotarget.

[81] Wong C.N., Fessas P., Dominy K., Mauri F.A., Kaneko T., Parcq P.D., Khorashad J., Toniutto P., Goldin R.D., Avellini C. (2021). Qualification of tumour mutational burden by targeted next-generation sequencing as a biomarker in hepatocellular carcinoma. Liver Int..

[82] Jhunjhunwala S., Hammer C., Delamarre L. (2021). Antigen presentation in cancer: Insights into tumour immunogenicity and immune evasion. Nat. Rev. Cancer.

[83] Rosenthal R., Cadieux E.L., Salgado R., Bakir M.A., Moore D.A., Hiley C.T., Lund T., Tanić M., Reading J.L., Joshi K. (2019). Neoantigen-directed immune escape in lung cancer evolution. Nature.

[84] Haber P.K., Castet F., Torres-Martin M., Andreu-Oller C., Puigvehí M., Miho M., Radu P., Dufour J.-F., Verslype C., Czauderna C. (2023). Molecular Markers of Response to Anti-PD1 Therapy in Advanced Hepatocellular Carcinoma. Gastroenterology.

[85] Hause R.J., Pritchard C.C., Shendure J., Salipante S.J. (2016). Classification and characterization of microsatellite instability across 18 cancer types. Nat. Med..

[86] Davoli T., Uno H., Wooten E.C., Elledge S.J. (2017). Tumor aneuploidy correlates with markers of immune evasion and with reduced response to immunotherapy. Science.

[87] Xu Y., Poggio M., Jin H.Y., Shi Z., Forester C.M., Wang Y., Stumpf C.R., Xue L., Devericks E., So L. (2019). Translation control of the immune checkpoint in cancer and its therapeutic targeting. Nat. Med..

[88] Shen J., Ju Z., Zhao W., Wang L., Peng Y., Ge Z., Nagel Z.D., Zou J., Wang C., Kapoor P. (2018). ARID1A deficiency promotes mutability and potentiates therapeutic antitumor immunity unleashed by immune checkpoint blockade. Nat. Med..

[89] Li J., Wang W., Zhang Y., Cieślik M., Guo J., Tan M., Green M.D., Wang W., Lin H., Li W. (2020). Epigenetic driver mutations in ARID1A shape cancer immune phenotype and immunotherapy. J. Clin. Investig..

[90] Zhou J., Liu M., Sun H., Feng Y., Xu L., Chan A.W.H., Tong J.H., Wong J., Chong C.C.N., Lai P.B.S. (2018). Hepatoma-intrinsic CCRK inhibition diminishes myeloid-derived suppressor cell immunosuppression and enhances immune-checkpoint blockade efficacy. Gut.

[91] Chiang D.Y., Villanueva A., Hoshida Y., Peix J., Newell P., Minguez B., LeBlanc A.C., Donovan D.J., Thung S.N., Solé M. (2008). Focal gains of VEGFA and molecular classification of hepatocellular carcinoma. Cancer Res..

[92] von Felden J., Craig A.J., Garcia-Lezana T., Labgaa I., Haber P.K., D’Avola D., Asgharpour A., Dieterich D., Bonaccorso A., Torres-Martin M. (2021). Mutations in circulating tumor DNA predict primary resistance to systemic therapies in advanced hepatocellular carcinoma. Oncogene.

[93] Thorsson V., Gibbs D.L., Brown S.D., Wolf D., Bortone D.S., Ou Yang T.-H., Porta-Pardo E., Gao G.F., Plaisier C.L., Eddy J.A. (2018). The Immune Landscape of Cancer. Immunity.

[94] Montal R., Sia D., Montironi C., Leow W.Q., Esteban-Fabró R., Pinyol R., Torres-Martin M., Bassaganyas L., Moeini A., Peix J. (2020). Molecular classification and therapeutic targets in extrahepatic cholangiocarcinoma. J. Hepatol..

[95] Job S., Rapoud D., Dos Santos A., Gonzalez P., Desterke C., Pascal G., Elarouci N., Ayadi M., Adam R., Azoulay D. (2020). Identification of Four Immune Subtypes Characterized by Distinct Composition and Functions of Tumor Microenvironment in Intrahepatic Cholangiocarcinoma. Hepatology.

[96] Gay C.M., Stewart C.A., Park E.M., Diao L., Groves S.M., Heeke S., Nabet B.Y., Fujimoto J., Solis L.M., Lu W. (2021). Patterns of transcription factor programs and immune pathway activation define four major subtypes of SCLC with distinct therapeutic vulnerabilities. Cancer Cell.

[97] Nsengimana J., Laye J., Filia A., O’Shea S., Muralidhar S., Poźniak J., Droop A., Chan M., Walker C., Parkinson L. (2018). β-Catenin-mediated immune evasion pathway frequently operates in primary cutaneous melanomas. J. Clin. Investig..

[98] Chen Y.-P., Wang Y.-Q., Lv J.-W., Li Y.-Q., Chua M.L.K., Le Q.-T., Lee N., Colevas A.D., Seiwert T., Hayes D.N. (2019). Identification and validation of novel microenvironment-based immune molecular subgroups of head and neck squamous cell carcinoma: Implications for immunotherapy. Ann. Oncol..

[99] Topalian S.L., Taube J.M., Anders R.A., Pardoll D.M. (2016). Mechanism-driven biomarkers to guide immune checkpoint blockade in cancer therapy. Nat. Rev. Cancer.

[100] Gibney G.T., Weiner L.M., Atkins M.B. (2016). Predictive biomarkers for checkpoint inhibitor-based immunotherapy. Lancet Oncol..

[101] Topalian S.L., Hodi F.S., Brahmer J.R., Gettinger S.N., Smith D.C., McDermott D.F., Powderly J.D., Carvajal R.D., Sosman J.A., Atkins M.B. (2012). Safety, activity, and immune correlates of anti-PD-1 antibody in cancer. N. Engl. J. Med..

[102] Rosenberg J.E., Hoffman-Censits J., Powles T., van der Heijden M.S., Balar A.V., Necchi A., Dawson N., O’Donnell P.H., Balmanoukian A., Loriot Y. (2016). Atezolizumab in patients with locally advanced and metastatic urothelial carcinoma who have progressed following treatment with platinum-based chemotherapy: A single-arm, multicentre, phase 2 trial. Lancet.

[103] Garon E.B., Rizvi N.A., Hui R., Leighl N., Balmanoukian A.S., Eder J.P., Patnaik A., Aggarwal C., Gubens M., Horn L. (2015). Pembrolizumab for the treatment of non-small-cell lung cancer. N. Engl. J. Med..

[104] Reck M., Rodríguez-Abreu D., Robinson A.G., Hui R., Csőszi T., Fülöp A., Gottfried M., Peled N., Tafreshi A., Cuffe S. (2016). Pembrolizumab versus Chemotherapy for PD-L1-Positive Non-Small-Cell Lung Cancer. N. Engl. J. Med..

[105] Sunshine J., Taube J.M. (2015). PD-1/PD-L1 inhibitors. Curr. Opin. Pharmacol..

[106] Pinato D.J., Mauri F.A., Spina P., Cain O., Siddique A., Goldin R., Victor S., Pizio C., Akarca A.U., Boldorini R.L. (2019). Clinical implications of heterogeneity in PD-L1 immunohistochemical detection in hepatocellular carcinoma: The Blueprint-HCC study. Br. J. Cancer.

[107] Zhou J., Cheung A.K., Liu H., Tan Z., Tang X., Kang Y., Du Y., Wang H., Liu L., Chen Z. (2013). Potentiating functional antigen-specific CD8^+^ T cell immunity by a novel PD1 isoform-based fusion DNA vaccine. Mol. Ther..

[108] Riaz N., Havel J.J., Makarov V., Desrichard A., Urba W.J., Sims J.S., Hodi F.S., Martín-Algarra S., Mandal R., Sharfman W.H. (2017). Tumor and Microenvironment Evolution during Immunotherapy with Nivolumab. Cell.

[109] Tumeh P.C., Harview C.L., Yearley J.H., Shintaku I.P., Taylor E.J.M., Robert L., Chmielowski B., Spasic M., Henry G., Ciobanu V. (2014). PD-1 blockade induces responses by inhibiting adaptive immune resistance. Nature.

[110] Topalian S.L., Drake C.G., Pardoll D.M. (2015). Immune checkpoint blockade: A common denominator approach to cancer therapy. Cancer Cell.

[111] Fridman W.H., Pagès F., Sautès-Fridman C., Galon J. (2012). The immune contexture in human tumours: Impact on clinical outcome. Nat. Rev. Cancer.

[112] Dudek M., Pfister D., Donakonda S., Filpe P., Schneider A., Laschinger M., Hartmann D., Hüser N., Meiser P., Bayerl F. (2021). Auto-aggressive CXCR6^+^ CD8 T cells cause liver immune pathology in NASH. Nature.

[113] Leslie J., Mackey J.B.G., Jamieson T., Ramon-Gil E., Drake T.M., Fercoq F., Clark W., Gilroy K., Hedley A., Nixon C. (2022). CXCR2 inhibition enables NASH-HCC immunotherapy. Gut.

[114] Ponziani F.R., Bhoori S., Castelli C., Putignani L., Rivoltini L., Del Chierico F., Sanguinetti M., Morelli D., Paroni Sterbini F., Petito V. (2019). Hepatocellular Carcinoma Is Associated with Gut Microbiota Profile and Inflammation in Nonalcoholic Fatty Liver Disease. Hepatology.

[115] Schwabe R.F., Greten T.F. (2020). Gut microbiome in HCC—Mechanisms, diagnosis and therapy. J. Hepatol..

[116] Dapito D.H., Mencin A., Gwak G.-Y., Pradere J.-P., Jang M.-K., Mederacke I., Caviglia J.M., Khiabanian H., Adeyemi A., Bataller R. (2012). Promotion of hepatocellular carcinoma by the intestinal microbiota and TLR4. Cancer Cell.

[117] Ma C., Han M., Heinrich B., Fu Q., Zhang Q., Sandhu M., Agdashian D., Terabe M., Berzofsky J.A., Fako V. (2018). Gut microbiome-mediated bile acid metabolism regulates liver cancer via NKT cells. Science.

[118] Yoshimoto S., Loo T.M., Atarashi K., Kanda H., Sato S., Oyadomari S., Iwakura Y., Oshima K., Morita H., Hattori M. (2013). Obesity-induced gut microbial metabolite promotes liver cancer through senescence secretome. Nature.

[119] Dizman N., Meza L., Bergerot P., Alcantara M., Dorff T., Lyou Y., Frankel P., Cui Y., Mira V., Llamas M. (2022). Nivolumab plus ipilimumab with or without live bacterial supplementation in metastatic renal cell carcinoma: A randomized phase 1 trial. Nat. Med..

[120] Buder-Bakhaya K., Hassel J.C. (2018). Biomarkers for Clinical Benefit of Immune Checkpoint Inhibitor Treatment-A Review from the Melanoma Perspective and Beyond. Front. Immunol..

[121] Thompson J.R., Menon S.P. (2018). Liquid Biopsies and Cancer Immunotherapy. Cancer J..

[122] Voong K.R., Feliciano J., Becker D., Levy B. (2017). Beyond PD-L1 testing-emerging biomarkers for immunotherapy in non-small cell lung cancer. Ann. Transl. Med..

[123] Khagi Y., Goodman A.M., Daniels G.A., Patel S.P., Sacco A.G., Randall J.M., Bazhenova L.A., Kurzrock R. (2017). Hypermutated Circulating Tumor DNA: Correlation with Response to Checkpoint Inhibitor-Based Immunotherapy. Clin. Cancer Res..

[124] Galle P.R., Foerster F., Kudo M., Chan S.L., Llovet J.M., Qin S., Schelman W.R., Chintharlapalli S., Abada P.B., Sherman M. (2019). Biology and significance of alpha-fetoprotein in hepatocellular carcinoma. Liver Int..

[125] Zhu A.X., Kang Y., Yen C., Finn R.S., Galle P.R., Llovet J.M., Assenat E., Brandi G., Pracht M. (2019). Ramucirumab after sorafenib in patients with advanced hepatocellular carcinoma and increased α-fetoprotein concentrations ( REACH-2 ): A randomised, double-blind, placebo-controlled, phase 3 trial. Lancet Oncol..

[126] Sun X., Mei J., Lin W., Yang Z., Peng W., Chen J., Zhang Y., Xu L., Chen M. (2021). Reductions in AFP and PIVKA-II can predict the efficiency of anti-PD-1 immunotherapy in HCC patients. BMC Cancer.

[127] Fessas P., Possamai L.A., Clark J., Daniels E., Gudd C., Mullish B.H., Alexander J.L., Pinato D.J. (2020). Immunotoxicity from checkpoint inhibitor therapy: Clinical features and underlying mechanisms. Immunology.

[128] Lee M.S., Ryoo B.Y., Hsu C.H., Numata K., Stein S., Verret W., Hack S.P., Spahn J., Liu B., Abdullah H. (2020). Atezolizumab with or without bevacizumab in unresectable hepatocellular carcinoma (GO30140): An open-label, multicentre, phase 1b study. Lancet Oncol..

[129] Rimola J., Díaz-González Á., Darnell A., Varela M., Pons F., Hernandez-Guerra M., Delgado M., Castroagudin J., Matilla A., Sangro B. (2018). Complete response under sorafenib in patients with hepatocellular carcinoma: Relationship with dermatologic adverse events. Hepatology.

